# miR-101/METTL3 axis induces autophagy by interrupting FOXG1/EIF3J-AS1 binding in gliomas

**DOI:** 10.1038/s41419-025-08285-6

**Published:** 2025-12-13

**Authors:** Yaping Yan, Shanshan Liu, Ailing Luo, Mansi Cai, Xiaohong Zhang, Xiaodan Liu, Yingyi Xu, Yabei Su, Siyi Zhang, Jianhua Liu, Xiaoping Liu

**Affiliations:** 1 Department of Hematology and Oncology, Guangzhou Women and Children’s Medical Center, Guangdong Provincial Clinical Research Center for Child Health, Guangzhou Medical University, Guangzhou, China; 2 Division of Birth Cohort Study, Guangzhou Women and Children’s Medical Center, Guangdong Provincial Clinical Research Center for Child Health, Guangzhou Medical University, Guangzhou, China; 3 Department of Anesthesiology, Guangzhou Women and Children’s Medical Center, Guangdong Provincial Clinical Research Center for Child Health, Guangzhou Medical University, Guangzhou, China

**Keywords:** CNS cancer, Mechanisms of disease

## Abstract

The role of autophagy in glioma remains controversial, with long non-coding RNAs (lncRNAs) playing a crucial role in its regulation. N6-methyladenosine (m6A) modification influences lncRNA expression and function. Specifically, lncRNA EIF3J-AS1 acts as an oncogene in glioma, yet the mechanisms driving its upregulation remain unclear. This study demonstrates that EIF3J-AS1 expression is significantly elevated in glioblastoma multiforme (GBM) compared to low-grade glioma (LGG) and normal brain tissue. RNA sequencing (RNA-seq) identified EIF3J-AS1 as a target of the tumor suppressor miR-101, with functional assays showing its role in promoting glioma cell proliferation, inhibiting autophagy, and enhancing tumorigenesis in vivo. Methylated RNA immunoprecipitation (MeRIP) and bioinformatics analyses confirmed m6A modification of EIF3J-AS1, which correlates positively with the m6A methyltransferase METTL3 in glioma tissues. Mechanistically, METTL3 promotes m6A-dependent binding of EIF3J-AS1 to the transcription factor FOXG1. RNA-seq screening further identified macrophage migration inhibitory factor (MIF), an autophagy-promoting gene, as a downstream target of both METTL3 and EIF3J-AS1. Functional validation revealed that the METTL3/EIF3J-AS1/FOXG1 axis suppresses autophagy via MIF downregulation. Conversely, miR-101-mediated suppression of METTL3 disrupts EIF3J-AS1-FOXG1 binding, restoring MIF expression and promoting autophagy. These findings highlight EIF3J-AS1 and METTL3 as potential therapeutic targets, with disruption of EIF3J-AS1-FOXG1 interactions representing a novel autophagy-modulating strategy for glioma treatment.

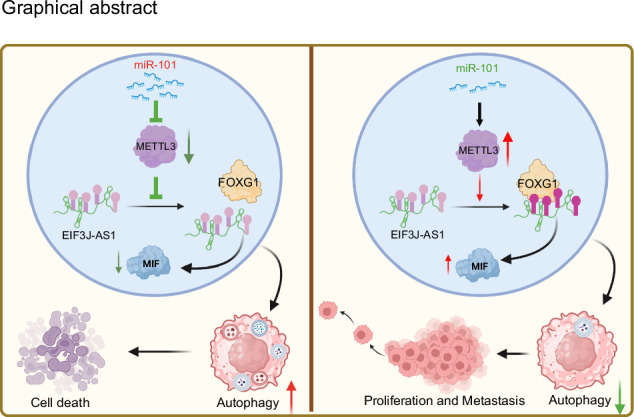

## Introduction

Gliomas account for ~80–85% of adult brain tumors, with a global incidence rate of 6 per 100,000 individuals annually [1]. Glioblastoma (GBM), the most aggressive glioma subtype, is the leading malignant brain tumor in adults [2]. Standard treatment includes surgical resection followed by radiotherapy and temozolomide chemotherapy; however, the median survival for GBM patients is only 15 months, with most experiencing relapse within 10 months [3]. There is an urgent need to elucidate glioma pathogenesis and develop more effective targeted therapies to improve patient outcomes.

Long non-coding RNAs (lncRNAs) are dysregulated in gliomas and contribute to disease progression. Given their high expression in brain tissue, lncRNAs are promising diagnostic markers and therapeutic targets [4]. EIF3J-AS1, an oncogenic lncRNA, promotes tumor proliferation and metastasis in multiple cancers [5]. It interacts with microRNAs (miRNAs), sponging miR-1343-3p to upregulate ANXA11 and drive glioma cell proliferation [6]. However, the mechanisms underlying EIF3J-AS1 upregulation in gliomas remain unclear.

RNA methylation is the most prevalent chemical modification observed in RNA molecules [7]. N6-methyladenosine (m6A) stands out as the predominant type of RNA methylation found in mammalian mRNAs and lncRNAs, exerting regulatory control over various aspects of RNA processing, including RNA production, splicing, translation, and transport [8]. The dynamic m6A modification process involves methyltransferases (METTL3, METTL14, WTAP), demethylases (FTO, ALKBH5), and RNA-binding proteins (YTH, HNRNP, and SRSF families) [9]. m6A-mediated RNA modifications influence glioma initiation and progression [10], with METTL3 upregulation promoting tumor progression via lncRNA modulation [11]. A previous study demonstrated that METTL3 induces LINC00475 splicing in an m6A-dependent manner, enhancing mitochondrial fission and autophagy to drive glioma progression. Additionally, RNA sequencing (RNA-seq) revealed that METTL3 overexpression elevates EIF3J-AS1 levels [12]. This study further investigates the impact of m6A transferase on EIF3J-AS1 expression and function in gliomas.

LncRNAs regulate gene expression by acting as competing endogenous RNAs (ceRNAs) for miRNAs, interfering with transcription factor binding, or recruiting epigenetic modifiers [13]. MiR-101, a known tumor suppressor, is downregulated in multiple cancers, inhibiting glioma cell proliferation [14], metastasis, and inducing apoptosis. Our previous work demonstrated that miR-101 promotes senescence [15] and modulates autophagy [16] in glioma cells. Mechanistically, miR-101 exerts tumor-suppressive effects by directly targeting or indirectly regulating lncRNAs [17], though the latter mechanism remains poorly understood. Recent study reported that METTL3 enhances the expression of miR-101-3p [18]. Whether miR-101 functions as a tumor suppressor via METTL3 and regulate the expression of METTL3 requires further investigation.

In this study, we will explore the mechanisms by which miR-101 and METTL3 regulate the expression of EIF3J-AS in glioma and its role in autophagy. By screening and identifying proteins that bind to EIF3J-AS, we will investigate whether METTL3 functions on the binding ability of EIF3J-AS with RNA-binding proteins in an m6A dependent manner. This will help clarify the role and regulatory mechanisms of EIF3J-AS in glioma cell autophagy. The findings will provide experimental evidence supporting m6A-targeted therapeutic strategies in gliomas.

## Materials and methods

### Databases

We obtained transcriptional and clinical data from the official website of the Chinese Glioma Genome Atlas (CGGA) (http://www.cgga.org.cn/), as well as from the TCGA database provided by the National Cancer Institute (https://tcga-data.nci.nih.gov/tcga/tcgaDownload.jsp). The m6A-modification sites of EIF3J-AS1 were predicted using the SRAMP database (http://www.cuilab.cn/sramp/).

### Cell lines

The human glioma cell lines (U87, U138, and U251) and HEK293T cells were obtained from Jennio (Guangzhou, China). All cell lines were routinely maintained and cultured in DMEM supplemented with 10% fetal bovine serum and 1% penicillin-streptomycin. The cultures were incubated at 37 °C in a CO_2_ incubator. Prior to the experiments, the cell lines were regularly validated using STR profiling and examined for mycoplasma infections.

### siRNA, mimics, vector constructs, and lentiviral particle production

The EIF3J-AS1 siRNAs were synthesized by RiboBio (Guangzhou, China), and MIF siRNAs were synthesized by Newhelix Biotechnology Co., Ltd. (Shanghai, China). The hsa-miR-101 mimic and negative control (NC) were procured from GenePharma (China). For the overexpression assay, a full-length human EIF3J-AS1 sequence was amplified by PCR and subcloned into the expression vector pcDNA3.1(+) to establish stable EIF3J-AS1-overexpressing cells. Glioma cells were transfected into 6-well plates using opti-mem and lipofectamine 3000 reagents as per the manufacturer’s instructions. Human METTL3 cDNA was successfully amplified from the U251 cell line and subsequently subcloned into the lentiviral expression vector designated as pHBLV-CMV-MCS-3flag-EF1-ZsGreen-T2A-PURO. This recombinant vector allows for the efficient expression of the METTL3 protein. In order to conduct a METTL3-knockout assay, two specific single-guide RNAs (shRNAs) were designed utilizing an online guide design tool (available at https://www.sigmaaldrich.cn/). These shRNAs were then meticulously cloned into another lentiviral expression vector, pHBLV-U6-MCS-CMV-ZsGreen-PGK-PURO, which facilitates the delivery of the guide RNA into the target cells. Following the construction of the lentiviral vectors, lentivirus was harvested 48 h after the co-transfection of the lenti-gRNA, pCMV-vsvg, and pSPAX2 vectors into HEK293T cells. The transfection process was achieved using Lipofectamine 2000, which enhanced the efficiency of the transfection and ultimately supported the production of high-titer lentivirus. This approach was deemed crucial for ensuing applications that require robust gene editing or expression analysis of METTL3. The related sequences used in the study are listed in Supplementary Table 1.

### RT-qPCR

The cell lines were subjected to the RNAiso Plus for total RNA isolation. The concentration of RNA was determined by measuring the absorbance at 260/280 nm using the Nanodrop Spectrophotometer. Subsequently, cDNAs were synthesized from the RNA using the PrimeScriptTM RT reagent Kit as per the manufacturer’s protocol. For quantitative reverse-transcription-PCR, the TB Green^®^ Premix Ex TaqTM II was employed. M-MLV (H-) Reverse Transcriptase and Hairpin-itTM miRNAs RT-PCR Quantitation Kit were used for quantifying the miRNA expression through quantitative reverse transcription. The relative changes in mRNA and the miRNA expression were calculated by using the 2^-ΔΔCt^ method with GAPDH and U6 serving as internal controls for normalization. Each sample was analyzed in triplicate. The primer sequences are listed in Supplementary Table 1.

### Cell counting kit-8 (CCK-8) assay

The cell viability was assessed using the CCK8 assay in accordance with the manufacturer’s instructions. Briefly, 1.0 × 10^3^ cells/well were cultured into a 96-well plate and analyzed at the specified time point using the CCK-8 kit. Each well was supplemented with 10 μL of the CCK-8 solution and incubated for 1 h at 37 °C. The absorbance was measured at a wavelength of 450 nm by using a microplate reader. Experiments were conducted at least thrice, and the representative data were presented.

### Cytoplasmic and nuclear protein extraction

The glioma cells were collected in trypsin-EDTA and centrifuged at 1000 rpm for 5 min. The cells were rinsed with PBS once and then centrifuged at 1000 rpm for 2–3 min. The supernatant was removed to collect the cell pellets and subjected to cytoplasmic and nuclear protein extraction according to the protocols of NE-PER Nuclear and Cytoplasmic Extraction Reagents, followed by reverse-transcribing of RNA samples to cDNA and quantification using the RT-qPCR assay. GAPDH was employed as a cytoplasmic control, while U6 served as a nuclear control. The 2^−ΔΔCt^ method was used to analyze the relative expressions of the genes in the nucleus and cytoplasm.

### Western blotting

The experimental cells were lysed on an ice bath for 20 min using RIPA buffer supplemented with protease inhibitor cocktails. Subsequently, the lysates were centrifuged at 14,000 rpm for 15 min. Protein quantification was performed using the Pierce BCA Protein Assay Kit, and the optical density was measured using the Spark Multimode microplate reader. An equal amount of proteins (20 µg) was separated on a 10% SDS-PAGE gel and transferred onto a PVDF membrane of 0.45-μm pore size. The membrane was then blocked in 5% skim milk at room temperature for 1 h before incubating overnight at 4 °C with a primary antibody. Following incubation, the membranes were washed thrice for 10 min each time in PBS supplemented with 0.1% Tween 20 and subsequently incubated with secondary antibodies at room temperature for 1 h. After washing, the blots were developed using the SuperECL Detection Reagent, and the images were acquired using the Chemidoc XRS+ system. Quantification analysis was performed utilizing ImageJ software. Information on antibodies for METTL3, MIF, FOXG1, LC3 I/II, p62, and GAPDH are provided in Supplementary Table 2.

### Autophagic flux assays

For autophagic flux assays, the mRFP-GFP-LC3 lentivirus was obtained from HANBIO (Shanghai, China). U251 cells infected with the mRFP-GFP-LC3 lentivirus were transfected with 2 μg of plasmids in a serum-free medium after 24 h. After a 48-h of incubation, the cells were fixed using 4% paraformaldehyde and subsequently stained with Hoechst for nuclear visualization under confocal laser microscopy. Autophagy was assessed by quantifying the percentage of LC3-positive cells, with a minimum count of 5 cells/group.

### m6A RNA immunoprecipitation (MeRIP) assay

Specific primer pairs targeting the predicted confidence m6A sites were designed for MeRIP PCR/qPCR analysis (Supplementary Table 1). Briefly, 300 μg of total RNA was randomly fragmented and then subjected to immunoprecipitation with anti-m6A antibody-coated Pierce™ Protein A/G Magnetic Beads at 4 °C for 2 h with rotation. The m6A-containing RNA fragments were enriched and subsequently eluted. The abundance of eluted RNA was quantified using PCR and RT-qPCR assays. The relative enrichment of m6A in the RNA segments was normalized to the input.

### RNA immunoprecipitation (RIP) assay

After 24 h of the transfection, a total of 2 × 10^7^ cells were collected for the RIP experiments by using an anti-FOXG1 antibody. Briefly, each group was lysed in 300 μL of the lysis buffer (100 mM KCl, 5 mM MgCl_2_, 10 mM HEPES [pH 7.0], 0.5% NP-40, 1 mM DTT, and 100 U/mL RNase inhibitor). The resulting supernatant was subjected to immunoprecipitation using protein A/G magnetic beads conjugated with FOXG1 antibody at a temperature of 4 °C overnight. Following the immobilization of the bead-bound complexes containing FOXG1 and the subsequent removal of unbound materials through three washes, proteins were digested using proteinase-K. The input and co-immunoprecipitated RNAs were extracted for qRT-PCR analysis as well as RNA-seq and agarose gel electrophoresis analyses.

### RNA-seq

The U251 cells were subjected to total RNA extraction using the RNAiso Plus kit according to the manufacturer’s protocol in a previous article [12]. Subsequently, 5 μg of RNA was used for RNA-seq analysis, and the quality assessment was performed using the Agilent 2100 Bioanalyzer. High-throughput RNA sequencing was conducted on the Illumina HiSeq X Ten Sequencing System. The differentially expressed genes (DEGs) were filtered based on a significant threshold value (P < 0.05) and fold change (log2|FC| ≥ 1).

### RNA pull-down assays

The biotinylated probe of EIF3J-AS1 was designed and synthesized by Gzscbio (Guangzhou, China). The sequence of the EIF3J-AS1 probe is provided in Supplementary Table S1. A pull-down assay was conducted using an RNA pull-down assay kit. Briefly, the beads were initially pretreated and incubated with the EIF3J-AS1 probe at 25 °C for 2–4 h to prepare streptavidin-labeled beads. Subsequently, total cell lysates containing Protease/Phosphatase Inhibitor Cocktail, and RNase inhibitor were incubated with either the EIF3J-AS1 probe or oligo probe at 4 °C overnight. After thorough washing thrice, the RNA complexes bound to the beads were eluted and extracted for mass spectrometry (MS) analysis or boiled in SDS buffer for Western blotting.

### Liquid chromatography-tandem mass spectrometry

The production of RNA pull-down was processed for LC-MS/MS analysis. The pull-down production was mixed with a solution of 8 M urea and 100 mM Tris-Cl and subjected to water bath sonication. After centrifugation, the supernatant was used for a reduction reaction (10 mM DTT, 37 °C for 1 h), followed by an alkylation reaction (40 mM iodoacetamide). Protein concentration was measured by the Bradford method. Urea was diluted below 2 M using 100 mM Tris-HCl (pH 8.0). Trypsin was added at a ratio of 1:50 (enzyme: protein, w/w) for overnight digestion at 37 °C. The next day, TFA was used to reduce the pH to 6.0 to end the digestion. The supernatant was subjected to peptide purification using the Sep-Pak C18 desalting column. The peptide eluate was vacuum-dried and stored at −20 °C for later use. LC-MS/MS data acquisition was performed on the Q Exactive plus mass spectrometer coupled with the Easy-nLC 1200 System. The specific parameters are described in a previous article [19].

### Animal experiments

Animal research was approved by the ethics committee of the Guangzhou Medical University (G2022-115) and was conducted in accordance with established guidelines and protocols for animal care and protection. BALB/c male nude mice (4-week-old) were obtained from Guangzhou Ruige Biological Technology Co., Ltd. (Guangzhou, China). U251 cells, tagged with a luciferase reporter, were transfected with either EIF3J-AS1-overexpressing plasmids or pcDNA3.1 plasmids. In addition, siEIF3J-AS1 or siNC were transfected into the U251-luciferase cell line, which stably expressed METTL3. These cells were subsequently subcutaneously injected into nude mice (average weight ~20 g, n = 5, 5 × 10^6^ cells per mouse). Data on the weight of nude mice and tumor volume were collected 10 days post-injection of U251 cells, with daily measurements taken up to 28 days. Following this step, D-luciferin (15 mg/mL) was injected into the mouse peritoneum at a dose of 10 μL/g, after which a 15-min wait was observed before placing the mice in a dark room for bioluminescence imaging. The tumor load was evaluated weekly using bioluminescence imaging, and the IVIS spectral imaging system (emission wavelength 510 nm) was employed for analysis. Ten minutes prior to in vivo imaging, the mice were anesthetized via inhalation of 3% isoflurane. After 28 days, the mice were euthanized with an injection of sodium pentobarbital, and the tumors were excised, photographed, and weighed.

### Polychromatic immunofluorescence (IF) and fluorescence in situ hybridization (FISH) staining of the glioma sections

The paraffin-embedded tissue microarrays, containing 76 glioma/3 adjacent non-carcinoma samples were constructed by Shanghai Xinchao Biotech (HBraG079PG01, Shanghai, China). The process of polychromatic IF staining was conducted by using a 4-color multiple fluorescent immunohistochemical staining kit, specifically designated as abs50012 and provided by Absin, a company based in Shanghai, China. This staining procedure was executed in accordance with the instructions provided by the manufacturer, leveraging the Tyramide signal amplification (TSA) technique, which enhances the sensitivity and specificity of the IF detection. For multiple fluorescent staining, the sections were processed beginning from the antigen-retrieval step to remove any bound antibodies, followed by incubation with an additional primary antibody before binding to the probe. This procedure was repeated until all antigens were stained. The relevant antibody information is provided in the Supplementary Table. The tissue sections were examined by employing the Panoramic P250-018007 scanning confocal microscope (Pannoramic DESK, Budapest, Hungary). For the acquisition of confocal images, objectives with either 20× or 10× magnifications were utilized, allowing detailed visualization of the tissue samples. The study was approved by the Ethics Committee on Scientific Research of Guangzhou Women and Children’s Medical Center (No. 117A01). All samples were collected with informed consent.

### Statistical analysis

The number of colonies and band intensity in Western blotting were quantified using ImageJ. Statistical analysis was performed using GraphPad Prism 9.0 software. Detailed statistical information is provided in the respective figure legends. Data were analyzed using a two-tailed unpaired Student’s *t*-test and presented as the mean ±SD. The cell proliferation curves were analyzed using two-way ANOVA. Kaplan–Meier survival curves were analyzed using the log-rank test. All experiments were performed a minimum of three times. Significance was defined as *P < 0.05, **P < 0.01.

### Ethical statement

The study was approved by the Ethics Committee on Scientific Research of Guangzhou Women and Children’s Medical Center (No. 117A01), Guangzhou Medical University, and informed consent was obtained from all participating patients in the study. All animal experiments were conducted in agreement with the Guide for the Care and Use of Laboratory Animals and were approved by the Institutional Animals Care and Use Committee of Guangzhou Medical University (No. G2022-115).

## Results

### EIF3J-AS1 is a potential prognosis marker and inhibits autophagy in glioma

**Fig. 1:**
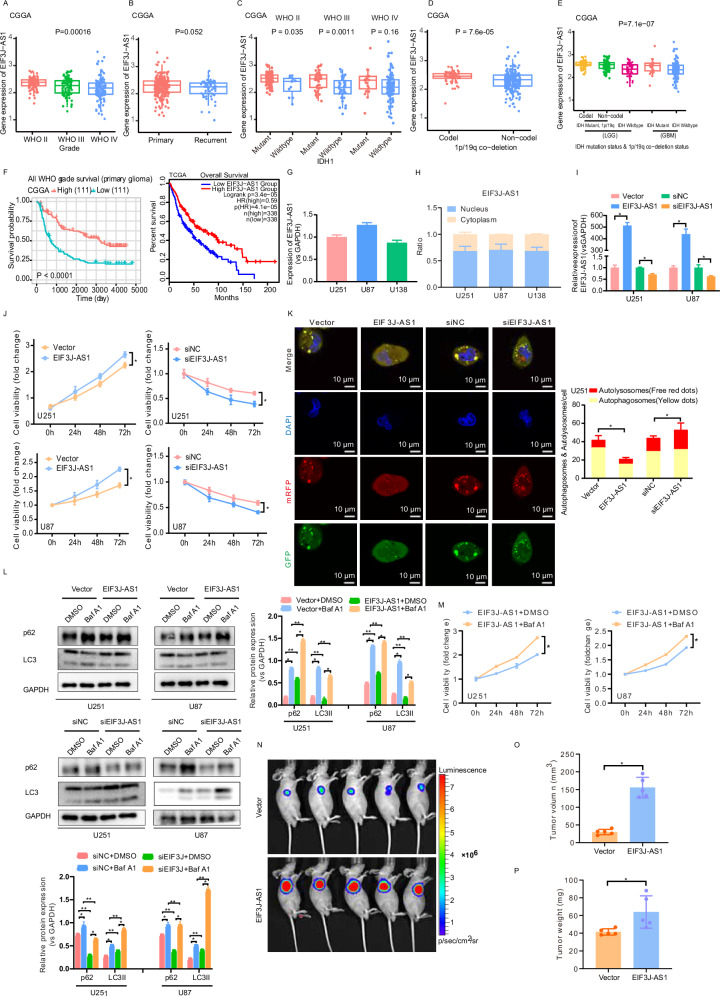
The expression and functions of EIF3J-AS1 in glioma. **A** The expression of EIF3J-AS1 was examined in different histological grades with reference to the CGGA databases. **B** The expression of EIF3J-AS1 in primary and recurrent gliomas was analyzed using CGGA databases. The expression of EIF3J-AS1 in patients stratified by the IDH-mutation status (**C**), 1p/19q co-deletion status (**D**), and joint analytical expression (**E**) with different WHO grades was also investigated using CGGA databases. **F** The correlation of EIF3J-AS1 expression with prognosis in primary glioma patients based on the CGGA dataset and the TCGA dataset. **G** The expression of EIF3J-AS1 in glioma cells was detected by RT-qPCR. GAPDH and U6 were used as positive controls. **H** RT-qPCR was performed to determine the presence of EIF3J-AS1 in the nuclear and cytoplasmic fractions of U251, U87, and U138 cell lysates. GAPDH and U6 served as positive controls. **I** RT-qPCR was used to assess the expression of EIF3J-AS1 transfected with EIF3J-AS1 overexpression plasmid or siRNA in U251 and U87 cells. **J** CCK-8 assays were employed to assess the viability of U251 and U87 cells transfected with EIF3J-AS1-overexpression plasmids or siRNA. **K** An image-based co-localization analysis of mRFP-GFP-LC3 was used to evaluate autophagy flux following miR-101 and/or EIF3J-AS1 overexpression in U251 cells, the nucleus was stained with DAPI (blue), GFP (green), mRFP (red). **L** Western blotting was conducted to examine LC3II expression in U251 and U87 cells transfected with EIF3J-AS1-overexpression plasmids or siRNA, with or without Baf A1 treatment, DMSO was used as vehicle control, with GAPDH serving as the internal control. **M** CCK-8 assays were employed to assess the viability of glioma cells transfected with EIF3J-AS1-overexpression plasmids and treated with Bafilomycin A1. **N** Representative images of in vivo fluorescence assays following the injection of luciferase-labeled U251 cells transfected with mock vector (n = 5) or EIF3J-AS1 overexpression plasmids (n = 5) on day 28 after tumor implantation, mice’s weight and tumor volume were weekly monitored for 4 weeks. **O** The tumor volumes of 4 weeks were compared. **P** The weight of each mouse was compared on week 4 after tumor implantation. The measurement data were presented as the mean ± SD. All tests in this study were repeated thrice. *P < 0.05, **P < 0.01.

To clarify the expression of EIF3J-AS1 in glioma patients, we analyzed the CGGA database showed that EIF3J-AS1 expression was highest in WHO grade II gliomas but decreased with increasing tumor grade (Fig. 1A). However, no significant difference was observed between primary and recurrent gliomas (Fig. 1B). Notably, EIF3J-AS1 expression was significantly higher in IDH-mutant gliomas compared to wild-type gliomas across all grades (Fig. 1C) and was elevated in patients with 1p/19q co-deletion compared to those without (Fig. 1D). In contrast, IDH1 wild-type low-grade gliomas and glioblastomas exhibited lower EIF3J-AS1 expression. Additionally, 1p/19q co-deletion status had no impact on EIF3J-AS1 expression in IDH-mutant gliomas (Fig. 1E). Survival analysis indicated that patients with high EIF3J-AS1 expression had better outcomes than those with low expression (Fig. 1F). Among glioma cell lines, EIF3J-AS1 expression was highest in U87 cells, followed by U251 cells (Fig. 1G). To investigate the function of EIF3J-AS1, we performed a nuclear-cytoplasmic fractionation assay to determine its subcellular localization in glioma cells. The results indicated that EIF3J-AS1 was predominantly localized in the nucleus but also expressed in the cytoplasm (Fig. 1H). Functionally, EIF3J-AS1 overexpression significantly enhanced the proliferation of U251 and U87 cells, whereas its knockdown significantly suppressed glioma cell proliferation (Fig. 1I, J). EIF3J-AS1 overexpression inhibited autophagy (Fig. 1K). To observe the effect of EIF3J-AS1 on autophagy flux, the expression of LC3II was detected. The overexpression of EIF3J-AS1 reduced the levels of LC3II. When in the presence of Baf A1, the expression of LC3II was significantly increased compared to the DMSO group, but it in EIF3J-AS1 overexpressing cells was still lower than that in the empty vector group; in EIF3J-AS1-silenced cells, the level of LC3II was noticeably increased compared to the empty vector group and the DMSO group (Fig. 1L). Additionally, EIF3J-AS1 overexpression promoted cell proliferation (Fig. 1M). In vivo, EIF3J-AS1 overexpression significantly enhanced tumorigenesis compared to the empty vector control (Fig. 1N–P).

### miR-101 promotes autophagy in gliomas by suppressing EIF3J-AS1 expression

**Fig. 2:**
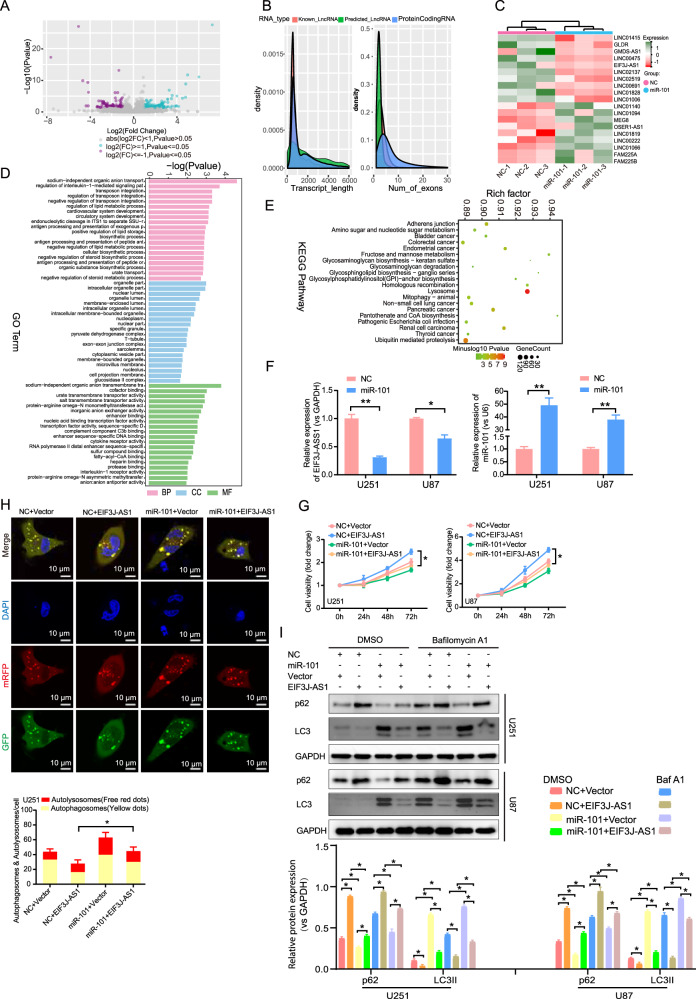
The effect of miR-101 on the expression and function of EIF3J-AS1 in gliomas. **A** Volcano plot illustrating the differential expression of lncRNAs in U251 cells with miR-101 overexpression, as determined by RNA-seq analysis, red dots represent upregulated genes, blue dots represent downregulated genes, and gray dots indicate no significant differential expression of genes. **B** The peak map displays the distribution of the length and exon number for differentially expressed lncRNA transcripts. **C** Heat maps depicted the upregulated or downregulated expression of lncRNAs influenced by miR-101. **D** GO analysis was employed to investigate the cellular processes associated with differentially expressed lncRNAs. **E** The KEGG pathway analyzed revealed signal transduction involving differentially expressed lncRNA road. **F** RT-qPCR was employed to assess the relative EIF3J-AS1 expressions in U251 cells transfected with miR-101 mimics. **G** CCK-8 assays were employed to assess the viability of glioma cells transfected with EIF3J-AS1-overexpression plasmids and miR-101 mimics. **H** Imaging of mRFP-GFP-LC3 infection in miR-101-overexpressed U251 cells transfected with overexpressing plasmids or siRNA of EIF3J-AS1. **I** The protein levels of LC3II and p62 were assessed in U251 and U87 cells transfected with miR-101 mimics plus EIF3J-AS1 overexpression plasmids, with or without Baf A1 treatment, DMSO was used as vehicle control, with GAPDH serving as the internal control. The measurement data are presented as the mean ± SD. All tests in this study were repeated thrice. *P < 0.05, **P < 0.01.

We performed RNA sequencing (RNA-seq) to identify differentially expressed lncRNAs modulated by miR-101. A total of 197 significantly dysregulated lncRNAs were identified, including 93 downregulated and 104 upregulated transcripts (Fig. 2A). Most had an approximate length of 1000 nucleotides and contained around five exons (Fig. 2B). Among the top 10 differentially expressed lncRNAs, EIF3J-AS1 was prominently identified (Fig. 2C). GO analysis indicated that these lncRNAs were primarily involved in intracellular metabolic processes and exhibited RNA and protein binding functions (Fig. 2D). KEGG analysis revealed their enrichment in multiple cancer-associated signaling pathways, as well as lysosomal and mitophagy pathways (Fig. 2E). Overexpression of miR-101 significantly downregulated EIF3J-AS1 expression in glioma cells (Fig. 2F). Moreover, co-overexpression of miR-101 and EIF3J-AS1 resulted in greater proliferation than miR-101 overexpression alone (Fig. 2G). Mechanistically, miR-101 promoted autophagy (Fig. 2H) by downregulating p62 and upregulating LC3II expression. However, restoration of EIF3J-AS1 in miR-101-overexpressing U251 cells significantly reduced autophagy, as evidenced by increased p62 levels and decreased LC3II levels (Fig. 2I). In the presence of Baf A1, miR-101 also could suppress p62 expression and enhance LC3II expression, while overexpression of EIF3J-AS1 could reverse the effect of miR-101 on the expression of p62 and LC3II (Fig. 2I). Collectively, these findings demonstrate that miR-101 suppresses glioma cell proliferation and promotes autophagy by downregulating EIF3J-AS1.

### METTL3 enhances the expression and function of EIF3J-AS1 via the m6A-dependent manner in gliomas

**Fig. 3:**
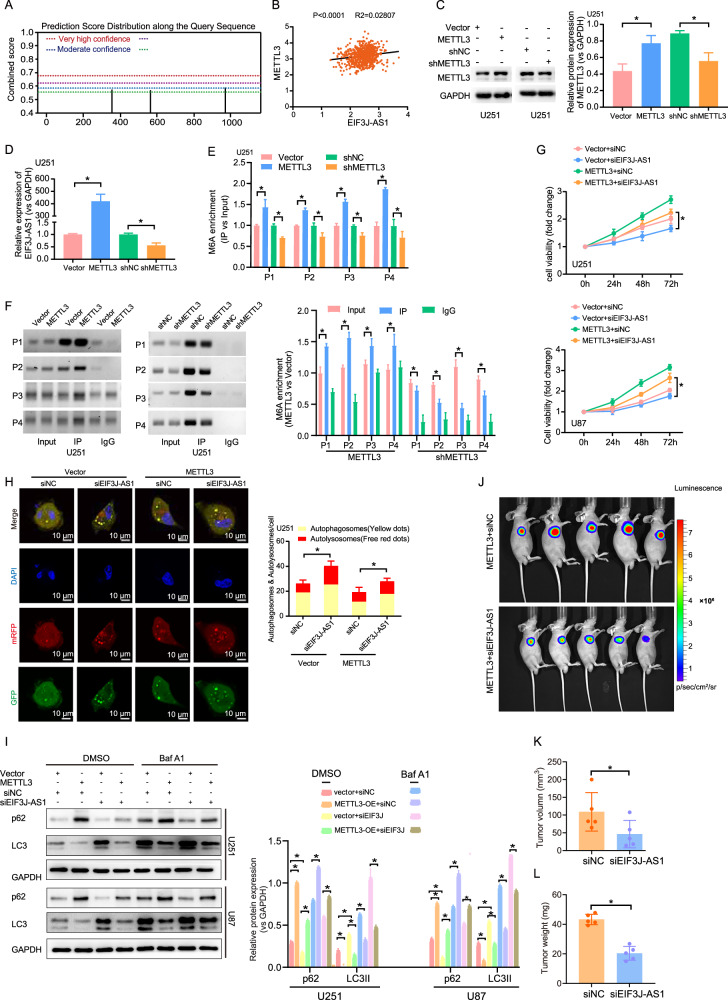
METTL3 upregulates the expression and function of EIF3J-AS1 through m6A modification. **A** Multiple m6A-methylation binding sites within the exon region of EIF3J-AS1 were predicted through the SRAMP database. **B** Correlation between the EIF3J-AS1 expression and METTL3 expression in the TCGA database. **C** The infection efficiency of METTL3 overexpression or knockdown lentivirus in U251 cells was assessed using Western blotting. **D** RT-qPCR assay revealed the expression of EIF3J-AS1 after infecting with METTL3 overexpression or shRNA lentivirus in U251 cells, with GAPDH as the internal control. MeRIP-qPCR (**E**) and agarose gel images of MeRIP-PCR (**F**) were used to evaluate the m6A enrichment of EIF3J-AS1 in U251 cells after METTL3 overexpression or shRNA lentivirus infection. **G** The proliferation was analyzed by CCK8 assay in U251 and U87 cells with METTL3-overexpression lentivirus infection plus EIF3J-AS1-knockdown plasmids transfection. **H** An image-based co-localization analysis of mRFP-GFP-LC3 was used to evaluate autophagy flux following METTL3 overexpression plus EIF3J-AS1 knockdown in U251 cells, and the nucleus was stained with DAPI (blue), GFP (green), and mRFP (red). **I** Western blotting revealed the expressions of LC3 II and p62 in U251 and U87 cells with METTL3 overexpression lentivirus infection plus EIF3J-AS1-knockdown plasmid transfection, with or without Baf A1 treatment, DMSO was used as vehicle control, using GAPDH as an internal control. **J** Representative images of in vivo fluorescence assays following the injection of METTL3-overexpressed luciferase-positive U251 cells transfected with siNC (n = 5) or siEIF3J-AS1 plasmids (n = 5) on day 28 after tumor implantation, and the mice’s weight and tumor volume were weekly monitored for 4 weeks. **K** The tumor volume of 4 weeks was also compared. **L** The weight of each mouse was compared on week 4 after the tumor implantation. The measurement data were presented as the mean ± SD. All tests in this study were repeated thrice. *P < 0.05, **P < 0.01.

As miR-101 was not predicted to directly bind to EIF3J-AS1, we used SRAMP to identify potential m6A modification sites within EIF3J-AS1 (Fig. 3A). Analysis of TCGA transcriptome data revealed a significant positive correlation between METTL3 and EIF3J-AS1 expression in glioma tissues (P < 0.001, R^2^ = 0.02807) (Fig. 3B). To investigate the regulatory role of METTL3, we overexpressed or silenced METTL3 in U251 cells (Fig. 3C). METTL3 overexpression significantly upregulated EIF3J-AS1 expression, whereas its knockdown led to a significant reduction in EIF3J-AS1 levels (Fig. 3D). Furthermore, MeRIP-qPCR analysis confirmed that METTL3 overexpression enhanced m6A enrichment on EIF3J-AS1, while METTL3 knockdown reduced m6A levels (Fig. 3E), a finding further validated by MeRIP-PCR gel electrophoresis (Fig. 3F). Functionally, METTL3 overexpression promoted glioma cell proliferation, but this effect was significantly attenuated when EIF3J-AS1 expression was suppressed (Fig. 3G). Additionally, METTL3 inhibited autophagy by upregulating p62 and downregulating LC3II. In METTL3-overexpressing glioma cells, EIF3J-AS1 knockdown led to decreased p62 and increased LC3II expression, thereby reversing METTL3-mediated autophagy suppression (Fig. 3H, I). With Baf A1 treatment, the expression of p62 in METTL3-overexpression and siNC group, and LC3II in empty vector and EIF3J-AS1-knockdown group was increased the most across all groups; suppression of EIF3J-AS1 could partially inhibit METTL3-induced p62 expression and restore the restriction of LC3II expression (Fig. 3I). In vivo, tumor formation experiments demonstrated that EIF3J-AS1 inhibition in METTL3-overexpressing cells significantly suppressed tumor growth (Fig. 3J–L). These findings suggest that METTL3 promotes EIF3J-AS1 expression by enhancing its m6A modification, thereby inhibiting autophagy in glioma cells.

### METTL3 obstructed autophagy-related gene MIF expression by enhancing EIF3J-AS1 expression

**Fig. 4:**
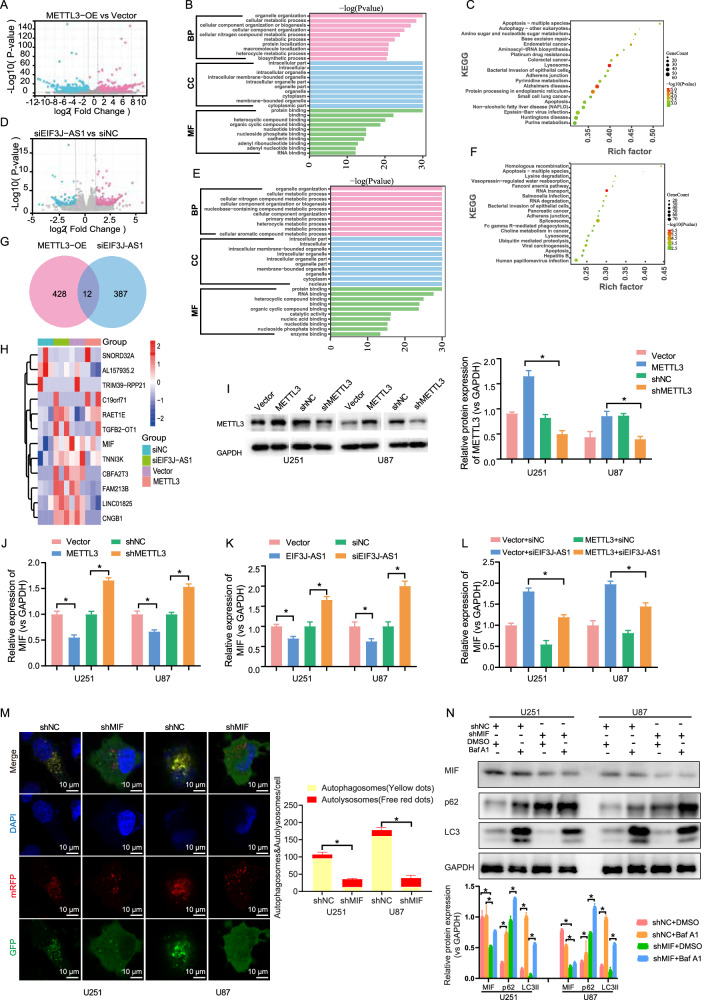
METTL3 inhibits autophagy-associated protein MIF by enhancing the EIF3J-AS1 expression in glioma. **A** Volcano plot illustrated the differential gene expression in U251 cells with METTL3 overexpression, as determined by RNA-seq assay. The red dots represent upregulated genes, the blue dots represent downregulated genes, and the gray dots indicate no significant differential expression of genes. GO (**B**) and KEGG (**C**) analysis of the DEG in the METTL3-overexpression group. **D** Volcano plot illustrated the differential gene expression in U251 cells with EIF3J-AS1 knockdown, as determined by RNA-seq assay. The red dots represent upregulated genes, the blue dots represent downregulated genes, and the gray dots indicate no significant differential expression of genes. GO (**E**) and KEGG (**F**) analysis of the DEG in the EIF3J-AS1 group. **G** The Venn diagram displayed significantly intersecting targets between the genes induced by METTL3 overexpression and those affected by the EIF3J-AS1 knockdown in U251 cells. **H** Heatmap depicts the expression of screened 12 mRNAs obtained from the intersection from (**G**). **I** Western blotting of the METTL3 expression in U251 and U87 cells with METTL3 overexpression or knockdown. The expression of MIF in U251 and U87 cells was analyzed by RT-qPCR after infection with lentivirus overexpressing or knocking-down METTL3 (**J**), transfection with EIF3J-AS1-overexpression plasmids and siRNA (**K**), and transfection with lentivirus overexpressing METTL3 plus plasmids knockdown of EIF3J-AS1 (**L**). **M** An image-based co-localization analysis of mRFP-GFP-LC3 was performed to evaluate autophagy flux following MIF knockdown in U251 cells. **N** Western blotting was performed to analyze the expression of LC3II in glioma cells infected with MIF shRNA lentivirus, with or without Baf A1 treatment, DMSO was used as vehicle control, using GAPDH as the internal control. The measurement data are presented as the mean ± SD. All tests in this study were repeated thrice. *P < 0.05.

To elucidate the mechanism by which METTL3 inhibits autophagy in gliomas via EIF3J-AS1, we performed RNA-seq to identify target genes regulated by METTL3. A total of 440 DEGs were identified, with 220 upregulated and 220 downregulated upon METTL3 overexpression (Fig. 4A). GO analysis indicated that these genes were primarily associated with organelles and exhibited nucleotide and RNA-binding functions (Fig. 4B). KEGG pathway analysis further revealed their enrichment in apoptosis, autophagy, and tumor signaling pathways (Fig. 4C). Similarly, RNA-seq analysis following EIF3J-AS1 inhibition identified 399 DEGs, including 211 upregulated and 188 downregulated genes (Fig. 4D). GO analysis showed their involvement in metabolic processes, organelle localization, and nucleotide, RNA, and protein binding (Fig. 4E). KEGG analysis indicated significant enrichment in RNA transport, degradation, splicing, and apoptosis pathways (Fig. 4F). To identify common target genes regulated by both METTL3 and EIF3J-AS1, we performed an intersection analysis, which yielded 12 shared genes, including SNORD32A, AL157935.2, TRIM39-RPP21, C19orf71, RAET1E, TGFB2-OT1, MIF, TNNI3K, CBFA2T3, FAM2138, LINC01725, and CNGB1 (Fig. 4G, H). MIF overexpression significantly improved autophagy [20]. We speculate whether METTL3 targets MIF through EIF3J-AS1 to inhibit autophagy in gliomas. RT-qPCR validation confirmed that METTL3 overexpression reduced MIF mRNA levels, whereas METTL3 inhibition increased its expression (Fig. 4I, J). Similarly, EIF3J-AS1 overexpression downregulated MIF mRNA levels, while its inhibition led to increased MIF expression (Fig. 4K). Notably, EIF3J-AS1 knockdown in METTL3-overexpressing glioma cells significantly rescued MIF expression (Fig. 4L). The inhibition of MIF could interrupt autophagy in U251 cells (Fig. 4M). Western blot analysis revealed that inhibition of MIF upregulated p62 expression and downregulated LC3II levels, whereas Baf A1 increased p62 levels and partially restored LC3II expression (Fig. 4N). Collectively, these findings suggest that METTL3 suppresses MIF expression through EIF3J-AS1, thereby inhibiting autophagy in glioma cells.

### miR-101 disturbed the binding of FOXG1 to EIF3J-AS1

**Fig. 5:**
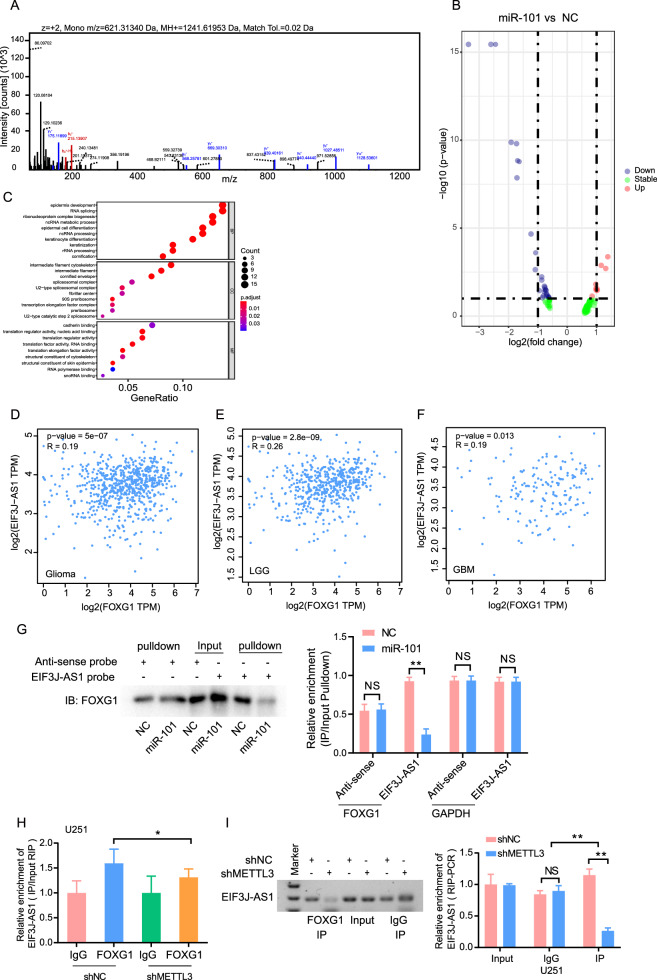
miR-101 interrupts the binding of FOXG1 to EIF3J-AS1. **A** Mass spectrometry identified FOXG1 peptides pulled down by the synthesized biotin-labeled EIF3J-AS1 probe in U251 cells transfected with miR-101 mimics. **B** Volcano plot illustrating DEGs in miR-101 overexpressed U251 cells by LC/MS, red dots: upregulated genes, blue dots: downregulated genes, gray dots: no significant differential expression genes. **C** GO was employed to analyze the significant binding proteins pulled down by the synthesized biotin-labeled EIF3J-AS1 probe. The scatter plots of TCGA indicate a correlation between the expression of EIF3J-AS1 and FOXG1 in glioma (**D**), GBM (**E**), and LGG (**F**). **G** RNA pull-down and Western blotting detected the interaction between EIF3J-AS1 and FOXG1 in U251 cells transfected with miR-101 mimics and enriched with a biotinylated EIF3J-AS1-specific probe. RIP assays were performed using anti-FOXG1 and anti-IgG antibodies in METTL3-knockdown U251 cells. The enrichments of EIF3J-AS1 by FOXG1 or IgG antibody were quantified via RT-qPCR (**H**) and agarose gel images (**I**). The measurement data were presented as the mean ± SD. All tests in this study were repeated thrice. *P < 0.05, **P < 0.01.

To investigate the mechanism by which miR-101 regulates EIF3J-AS1 expression, we performed pulldown-LC/MS to identify proteins that interact with EIF3J-AS1 (Fig. 5A). The analysis revealed that miR-101 overexpression in glioma cells enhanced the binding affinity of EIF3J-AS1 to three proteins while reducing its affinity for eleven proteins (Fig. 5B). These proteins are primarily involved in RNA splicing, ncRNA processing, and RNA-binding functions (Fig. 5C). Analysis of TCGA data demonstrated a positive correlation between FOXG1 and EIF3J-AS1 expression in glioma tissues (R = 0.19, P = 5 × 10^−7^) (Fig. 5D), as well as in GBM tissues (R = 0.19, P = 0.013) (Fig. 5E), with a stronger correlation observed in LGG tissues (R = 0.26, P = 2.8 × 10^−9^) (Fig. 5F). Pulldown-IP validation confirmed the interaction between FOXG1 and EIF3J-AS1, which was significantly weakened in miR-101-overexpressing cells compared to the control group (Fig. 5G). Moreover, METTL3 inhibition significantly disrupted the interaction between FOXG1 and EIF3J-AS1 (Fig. 5H, I), suggesting that miR-101 interferes with this binding, whereas METTL3 may facilitate it.

### FOXG1 participates in METTL3-targeted inhibition of autophagy by MIF

**Fig. 6:**
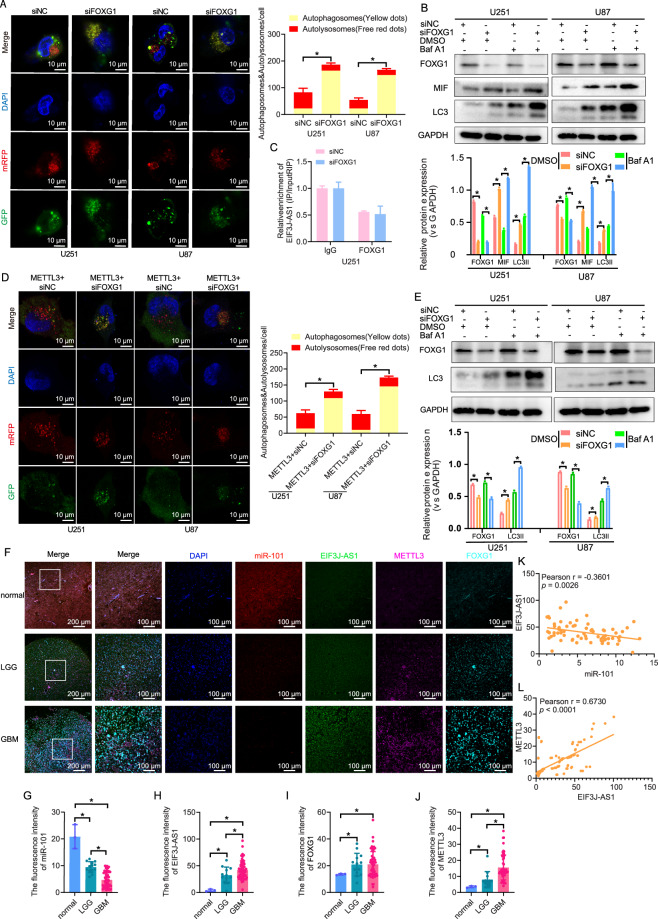
FOXG1 is involved in the inhibition of MIF by METTL3 in glioma cells. **A** An image-based co-localization analysis of mRFP-GFP-LC3 was performed to evaluate autophagy flux following FOXG1 knockdown in U251 and U87 cells. **B** Western blotting was performed to evaluate the expression of LC3II and MIF in U251 and U87 cells with FOXG1 knockdown, with or without Baf A1 treatment, DMSO was used as vehicle control. **C** The enrichments of EIF3J-AS1 by FOXG1 or IgG antibody were quantified via RIP-qPCR. **D** An image-based co-localization analysis of mRFP-GFP-LC3 was performed to evaluate autophagy flux following FOXG1 knockdown in METTL3-overexpressed U251 and U87 cells. **E** Western blotting was performed to evaluate the expression of LC3II and MIF in METTL3-overexpressed U251 and U87 cells with FOXG1 knockdown, with or without Baf A1 treatment, DMSO was used as vehicle control. **F** The expression of EIF3J-AS1 (green), METTL3 (purole), FOXG1 (cyan), and miR-101 (red) in gliomas and normal brain tissues was detected by FISH and IF multicolor staining. The differential expression of miR-101 (**G**), EIF3J-AS1 (**H**), FOXG1 (**I**) and METTL3 (**J**) was statistically analyzed among normal brain tissues, LGG and HGG. **K** The correlation between EIF3J-AS1 and miR-101. **L** The correlation between METTL3 and EIF3J-AS1. The measurement data are presented as the mean ± SD. All tests in this study were repeated thrice. *P < 0.05.

To determine whether FOXG1 is involved in METTL3/EIF3J-AS1-mediated autophagy suppression in glioma cells, we conducted a series of functional assays. FOXG1 knockdown significantly increased autophagic flux (Fig. 6A) and upregulated LC3II expression (Fig. 6B). MIF expression was significantly elevated following FOXG1 inhibition. Additionally, after Baf A1 treatment, there was no difference of MIF expression in FOXG1-silenced group with or without Baf A1 treatment; however, LC3II expression was remarkably elevated in FOXG1-silenced glioma cells compared to the other groups (Fig. 6B). However, RIP assays indicated that FOXG1 knockdown did not affect its binding to EIF3J-AS1 (Fig. 6C). Next, we examined the effects of FOXG1 inhibition in METTL3-overexpressing glioma cells. The siFOXG1 group exhibited significantly enhanced autophagic flux compared to METTL3 overexpression alone (Fig. 6D), accompanied by increased LC3II levels. Baf A1 has no effect on the expression of FOXG1. However, Baf A1 could significantly upregulate LC3II levels in FOXG1-silenced cells (Fig. 6E). To further assess expression patterns, we performed FISH and IF multicolor staining to analyze EIF3J-AS1, METTL3, FOXG1, and miR-101 in gliomas and normal brain tissues (Fig. 6F). miR-101 expression was significantly lower in gliomas, with the lowest levels detected in GBM (Fig. 6G). Conversely, EIF3J-AS1 expression was negligible in normal brain tissues but significantly upregulated in GBM compared to LGG (Fig. 6H). While FOXG1 expression was significantly elevated in gliomas compared to normal brain tissues, no significant difference was observed between GBM and LGG groups (Fig. 6I). METTL3 levels were significantly higher in GBM than in LGG and normal tissues (Fig. 6J). Correlation analysis confirmed that in glioma, EIF3J-AS1 expression was negatively correlated with miR-101 protein levels (r = −0.3601, P = 0.0026) (Fig. 6K) and positively correlated with EIF3J-AS1 expression (r = 0.6730, P < 0.0001) (Fig. 6L). These findings suggest that EIF3J-AS1 and METTL3 may serve as potential therapeutic targets for gliomas, with FOXG1 playing a key role in METTL3-induced inhibition of autophagy.

### miR-101 impedes the binding of FOXG1 to EIF3J-AS1 through inhibiting METTL3

**Fig. 7:**
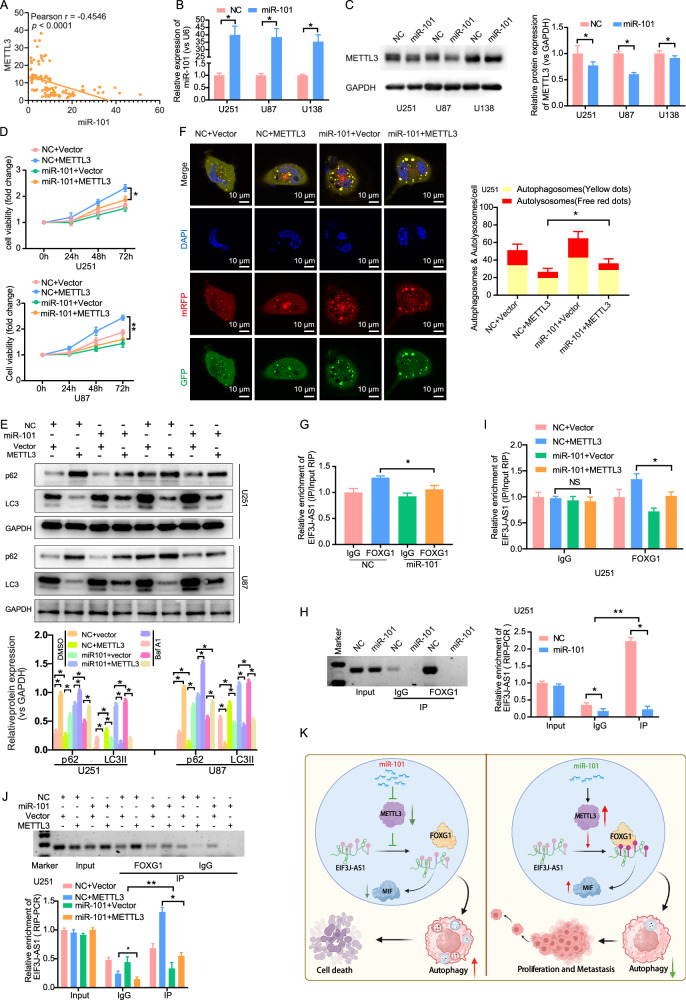
miR-101 reduces glioma cells autophagy by targeting METTL3-mediated FOXG1/EIF3J-AS1 binding. **A** The scatter plots depicting the correlation between the expression of miR-101-3p and METTL3 protein in tissue array. **B** U251, U87, and U138 cells were transfected with miR-101 mimics, and NC was detected by RT-qPCR. **C** The METTL3 protein expression was detected by Western blotting. **D** The proliferation capacity of U251 and U87 cells after the co-overexpression of miR-101 and METTL3 were evaluated by the CCK-8 assay. **E** Western blotting was performed to evaluate the expression of LC3II and p62 in U251 and U87 cells with the co-overexpression of miR-101 and METTL3, with or without Baf A1 treatment, DMSO was used as vehicle control. **F** Autophagy assay using mRFP-GFP-LC3 in U251 cells with the co-overexpression of miR-101 and METTL3. RIP assays were performed using anti-FOXG1 or anti-IgG antibodies in U251 cells with miR-101 overexpressing or co-overexpressing with FOXG1. The enrichment of EIF3J-AS1 was detected by RT-qPCR (**G**) and agarose gel images (**H**). The enrichment of EIF3J-AS1 was detected in U251 cells with the co-overexpression of miR-101 and METTL3 by RT-qPCR (**I**) and agarose gel images (**J**). **K** Schematic diagram of the molecular mechanism. The measurement data are presented as the mean ± SD. All tests in this study were repeated thrice. *P < 0.05, **P < 0.01.

To investigate whether miR-101 induces autophagy in glioma cells by disrupting the binding of FOXG1 to EIF3J-AS1 via METTL3 inhibition, we performed multicolor staining of a tissue array. A significant negative correlation was observed between miR-101 and METTL3 protein expression (r = −0.4546, P < 0.0001) (Fig. 7A). Overexpression of miR-101 in glioma cells (Fig. 7B) significantly suppressed METTL3 expression (Fig. 7C). Compared to the miR-101 overexpression group, glioma cells co-expressing miR-101 and METTL3 exhibited enhanced proliferation (Fig. 7D), restored p62 protein levels, decreased LC3II expression (Fig. 7E), and suppressed autophagic flux (Fig. 7F). Compared with DMSO, Baf A1 did not significantly elevate the expression levels of p62 in the METTL3 overexpression group or the miR-101 and METTL3 double overexpression group. METTL3 exerted an inhibitory effect on the LC3II expression induced by the combined treatment of Baf A1 and miR-101 (Fig. 7E). RIP assays further demonstrated that miR-101 overexpression significantly inhibited the binding of FOXG1 to EIF3J-AS1 (Fig. 7G, H). Notably, METTL3 overexpression partially restored the FOXG1-EIF3J-AS1 interaction in miR-101-overexpressing U251 cells, as confirmed by RIP assays (Figs. 7I, J). These findings suggest that EIF3J-AS1 is significantly upregulated in gliomas, where it promotes cell proliferation and inhibits autophagy. METTL3 facilitates the binding of EIF3J-AS1 to the transcription factor FOXG1 in an m6A-dependent manner, thereby enhancing EIF3J-AS1 expression. Additionally, METTL3 suppresses MIF expression, leading to autophagy inhibition. Conversely, the tumor-suppressive microRNA miR-101 inhibits EIF3J-AS1 expression by targeting METTL3, thereby inducing autophagy in glioma cells (Fig. 7K).

## Discussion

In this study, we identified miR-101 as an inhibitor of the m6A methyltransferase METTL3, which downregulates EIF3J-AS1 expression in an m6A modification-dependent manner. This process disrupts the binding of FOXG1 to EIF3J-AS1, induces MIF mRNA expression, and ultimately promotes autophagy in glioma cells.

Autophagy is a fundamental cellular process that maintains homeostasis by degrading, recycling, and removing damaged organelles, macromolecules, and misfolded proteins. Under normal physiological conditions, autophagy is tightly regulated; however, under stress conditions such as cell death, starvation, oxidative stress, and invasion, its activity is significantly enhanced. In glioma, autophagy exerts a dual role: it suppresses tumor progression by eliminating damaged organelles, reactive oxygen species, inflammation, and necrosis, but it can also promote tumorigenesis by recycling cellular components to sustain tumor metabolism and viability [21]. Notably, LC3-II expression is significantly higher in GBM than in LGG, and increased LC3 expression correlates with improved prognosis in GBM [22]. Accumulation of p62 promotes oxidative stress, tumor initiation, proliferation, and migration, while autophagy counteracts these effects by reducing p62 expression [23]. Additionally, MIF inhibitor treatment has been shown to suppress autophagy in airway remodeling [24], and MIF plays a critical role in autophagy regulation during carcinogenesis [25]. Our study confirmed that miR-101 enhanced autophagic flux in glioma cells by suppressing p62 levels and increasing the LC3II/I ratio. Furthermore, METTL3 suppresses MIF expression by upregulating EIF3J-AS1, thereby inhibiting autophagy in glioma cells.

METTL3 is the central catalytic subunit of the m6A methyltransferase complex, which mediates m6A modifications on RNA to regulate gene expression and cellular behaviors [26]. Elevated METTL3 expression has been observed in various tumors, contributing to increased m6A modification on target genes and promoting cancer progression. Notably, METTL3 overexpression is closely linked to glioma clinical stage, and its inhibition reduces m6A enrichment on splicing factor SRSF proteins, thereby decreasing their stability [27]. Targeted METTL3 inhibition has been shown to suppress mitophagy in glioma cells, thereby facilitating GBM stem cell proliferation and self-renewal and counteracting temozolomide-induced drug resistance [28]. Multiple mechanisms regulate METTL3 expression, including gene activity, transcription initiation, transcriptional modification, transport, and post-translational modifications [29]. miRNAs play a crucial role in METTL3 regulation: for instance, miR-302 inhibits METTL3 accumulation in M1 macrophages by targeting its 3’UTR region [30], while circVMP1 reduces METTL3 levels in NSCLC by sponging miR-524-5p [31]. In this study, we demonstrated that miR-101 facilitated autophagy by targeting METTL3 in glioma cells. However, direct binding of miR-101 to METTL3 was not observed, and further research is needed to elucidate the underlying regulatory mechanism.

METTL3 exerts oncogenic effects by modulating non-coding RNA expression and function. Specifically, METTL3 modifies ncRNAs via m6A modification, thereby regulating RNA Pol II transcription and promoting non-small cell lung cancer [32]. Additionally, METTL3 stabilizes the super-enhancer lncRNA SUCLG2-AS1, facilitating the formation of a long-range chromatin loop between the SOX2 enhancer and promoter, which promotes nasopharyngeal carcinoma metastasis and radiation insensitivity [33]. In colorectal cancer, m6A-modified circular RNA QSOX1 induces regulatory T cells, contributing to anti-CTLA4 resistance [34]. Moreover, METTL3 enhances the stability of lncRNA THAP7-AS1 in an m6A-dependent manner, promoting CUL4B nuclear translocation and tumorigenesis [35]. Our findings revealed that miR-101 suppressed EIF3J-AS1 expression and function in glioma cells by reducing METTL3-mediated m6A modification of EIF3J-AS1.

LncRNAs play an important role in regulating autophagy in glioma cells [36]. EIF3J-AS1 functions as a competitive endogenous RNA (ceRNA) by sponging miRNAs, thereby exerting an oncogenic role in multiple cancers. For instance, it promotes nasopharyngeal carcinoma invasion by sponging miR-373-3p and upregulating g AKT1 expression [37]. Additionally, H3K27 acetylation induces EIF3J-AS1 expression, leading to enhanced proliferation and suppressed apoptosis in colorectal cancer [38]. In our study, we found that EIF3J-AS1 overexpression inhibits autophagy in glioma cells by increasing MIF expression, thereby promoting glioma progression. Interestingly, high EIF3J-AS1 expression was positively correlated with favorable prognosis in glioma patients. This may be attributed to the elevated EIF3J-AS1 levels specifically observed in IDH-mutated and 1p/19q co-deleted gliomas. Patients with IDH-mutant gliomas generally exhibit better prognoses than those with IDH-wildtype counterparts [39] and 1p/19q co-deletion is exclusive to IDH-mutant gliomas [40]. Overall, our findings suggest that EIF3J-AS1 may serve as both a potential therapeutic target and a prognostic biomarker for glioma.

m6A modifications frequently occur near the stop codon and are dynamically regulated by three classes of regulatory proteins, which modify lncRNAs to promote glioma progression [41]. METTL3 activates the Wnt/β-catenin signaling pathway by promoting m6A methylation of LINC00839, thereby maintaining glioma stem cell stemness and inducing radioresistance [42]. Similarly, m6A enrichment in LINC01003 enhances glioma cell migration via the CAV1/FAK pathway [43]. Additionally, m6A modification on LINREP promotes GBM progression by recruiting the PTBP1/HuR complex [44], while m6A upregulation of WEE2-AS1 increases RPN2 stability, further facilitating GBM progression [45]. METTL3 also enhances MALAT1 stability through its interaction with RNA-binding protein HuR, leading to NF-κB activation and promoting IDH wild-type glioma progression [46]. These findings suggest that METTL3-mediated m6A modification influences the binding affinity between RNA-binding proteins and lncRNAs. In this study, we used pulldown-LC/MS to screen and confirmed that miR-101 disrupted the FOXG1–EIF3J-AS1 interaction. Moreover, METTL3 overexpression significantly restored the binding affinity between FOXG1 and EIF3J-AS1, counteracting the effect of miR-101.

M6A modification is reported to effect the binding of transcriptor and mRNA [47]. FOXG1, a key member of the forkhead family of nuclear transcription factors, plays a crucial role in development, differentiation, and cell survival across various tissues. Additionally, FOXG1 modulates autophagy by regulating mitochondrial function [48, 49]. Studies indicate that FOXG1 upregulation contributes to glioma progression, enhancing neural stem cell properties in GBM by regulating the cell cycle [50]. Furthermore, FOXG1 cooperates with Wnt/β-catenin signaling to activate GBM stem cells [51]. Additionally, FOXG1 induces radioresistance by enhancing autophagy in glioma cells [52]. Further investigations are needed to determine the specific FOXG1 domain that binds to EIF3J-AS1.

In conclusion, this study identified EIF3J-AS1 as a potential prognostic marker in glioma, as its high expression was associated with a favorable prognosis, particularly in patients with IDH mutations and 1p/19q co-deletions. miR-101 suppressed EIF3J-AS1 expression in glioma cells, while EIF3J-AS1 overexpression inhibited autophagy. Furthermore, miR-101 reduced m6A enrichment on EIF3J-AS1 by suppressing METTL3, thereby disrupting FOXG1–EIF3J-AS1 binding and inducing MIF expression, leading to autophagy activation and glioma suppression. Overall, these findings suggest that EIF3J-AS1 could serve as both a prognostic biomarker and a therapeutic target for glioma, providing new insights into disruption of EIF3J-AS1-FOXG1 interactions as a novel autophagy-modulating strategy for glioma treatment. Furthermore, this study also supplies experimental evidence for the treatment of glioma based on m6A modification editing.

## Supplementary information

original+data

Supplementary table

## Data Availability

The RNA-seq data reported in this study have been deposited in the Genome Sequence Archive (Genomics, Proteomics & Bioinformatics 2021) at the National Genomics Data Center (Nucleic Acids Res 2022), which is a part of the China National Center for Bioinformation/Beijing Institute of Genomics, Chinese Academy of Sciences. These data are publicly accessible at https://ngdc.cncb.ac.cn/gsa-human. The protein profile data reported in this paper have been deposited in OMIX, also under the auspices of the China National Center for Bioinformation/Beijing Institute of Genomics, Chinese Academy of Sciences (accession available at: https://ngdc.cncb.ac.cn/omix). All remaining data are presented within the article and the Supplementary Information Files and can be obtained from the corresponding author upon request.
