# Supplementary material for: miR-101/METTL3 axis induces autophagy by interrupting FOXG1/EIF3J-AS1 binding in gliomas
Source: Cell Death Dis. 2025 Dec 13;17(1):99. doi: 10.1038/s41419-025-08285-6 (PMC12830681; doi:10.1038/s41419-025-08285-6)
Supplement: Supplementary file 1 — original+data [file 41419_2025_8285_MOESM1_ESM.pdf]

Supplementary Table 1. List of primers, shRNA, siRNA, mimics and probeused in this study

qRT-PCR			
Gene Name	species	Forward	Rerverse
EIF3J-AS1	Homo sapiens	TGCCCTCCATCAAGTAGCAA	TTCAGTTTCAACCAGCGCCT
MIF		CTGCACAGCATCGGCAAGAT	AGTTGTTCCAGCCCACATTG
METTL3		TTCCGGTTAGCCTTCGGGG	CTGGGCTGTCACTACGGAAG
miR-101		CATCGCACGTACAGTACTGTGATA	CTCTGTCTCTCGTCTTGTTGGTAT
U6		CGCTTCGGCAGCACATATAC	TTCACGAATTTGCGTGTCATC
GAPDH		AGAAGGCTGGGGCTCATTTG	AGGGGCCATCCACAGTCTTC

shRNA or gRNA	species	Forward	Rerverse
shNC	Homo sapiens	GATCCAACAAGATGAAGAGCACCAATCAAGAGTTGGTGCTC TTCATCTTGTTGTTTTTG	AATTCAAAAACAACAAGATGAAGAGCACCAACTCTTGAT TGGTGCTCTTCATCTTGTTG
shMETTL3		GATCGCTGCACTTCAGACGAATTATTCAAGAGATAATTCGTC TGAAGTGCAGCTTTTTG	AATTCAAAAAGCTGCACTTCAGACGAATTATTCAAGAGAT AATTCGTCTGAAGTGCAGC

siRNA	species	Forward	Forward
NC		UUCUCCGAACGUGUCACGUTT	ACGUGACACGUUCGGAGAATT
siEIF3J-AS1	Homo sapiens	GGAAAGAUCUGUAGGAAUAAU	UAUUCCUACAGAUCUUUCCUG
		GGAACAGAGUCUACAAAUAAA	UAUUUGUAGACUCUGUUCCAU
		AGAUGGACAUGUAAACAAAUU	UUUGUUUACAUGUCCAUCUUU
siMIF		CCGAUGUUCAUCGUAAACACC	UGUUUACGAUGAACAUCGGCA
		CGGACAGGGUCUACAUCAACU	UUGAUGUAGACCCUGUCCGGG
		GGGUCUACAUCAACUAUUATT	UAAUAGUUGAUGUAGACCCTT

mimics	species	Forward	Rerverse
hsa-miR-101mimics	Homo sapiens	UACAGUACUGUGAUAACUGAA	CAGUUAUCACAGUACUGUAUU
hsa-mimics NC		UUCUCCGAACGUGUCACGUTT	ACGUGACACGUUCGGAGAATT

PCR

Gene Name	species	Forward	Rerverse
MIF	Homo sapiens	CCGGAATTCGTGGTGTCCGAGAAGTCAGG	CCCTCGAGTTCTCCCCACCAGAAGGTTG
METTL3		TCTACTAGAGGATCTATTTCCGGTGGCGTGAGAATTGGCTATAT	CTTCTAGAACTAGTCTCGAGGAATTCTATAAATTCTTAGGTTTAGAGATG
EIF3J-AS1		CCGGATATCCGCCGTGCTCAGAAG	TTGCGGCCGCATTTCTCTGGGTTGTTTTTTGTT

Primers used in MERIP

Gene Name	species	Forward	Rerverse
P1	Homo sapiens	GGGGCTGAGGAGTGCTGGGTC	GCGACGAAGACTGGCGAGGTT
P2		CGGAGGATGAAGGAAGGC	CGGCCCTGATCCACCTTT
P3		GGAAGGAGGCAAGAAAG	CGCCTAGCCAAGTTGAT
P4		CGATGCTACCCATGTTACTC	ACTGAGTAACATGGGTAGCATCGCC

EIF3J-AS1-sense RPD probe

Gene Name	species	Target sequence
EIF3J-AS1-sense	Homo sapiens	CGCCGTGCTCAGAAGAGGGCTCTGAGGCCACGCCCGCCTGTGGGGTCGGTGGCCGGGCTTCCGGGACCCCAGAGGGAGTC<br>GTCGCGCGCTAGGTGGAGGGGACCCCTCTGGAATCCGGCGATCCCCGGGCGCAGATGCAGGAGCGGGTGGGGAGAGCCGC<br>GTTCCTGGCATGTTTGCCTCGAGTTAGTCACGAAGGAATCGAAGAGAGCCTTAGTCTTCGAGGCTTTTCCCTTCCTGTGAGCC<br>CCTTTGAGGCCAGCACATTGATTTTTTTTTCTTTCGCAGAACAATTTCGGACCTCCTGGGGTCTATGCTAGGTATGAGGTGTAT<br>CTTTGCCCTCCATCAAGTAGCAAAGATGGACATGTAAACAAATTTCGACACTGTGATGACTGCCACAAAAAGCAGCATTATG<br>AGGTTGAATGTAAAAGGATAACCTTAGCGGCGGCGGGGGCCGGAGGATGAAGGAAGGCTTCCTGAGGTAGGCGCTGGTTGA<br>AACTGAAGTTGGAGAGAGCAAGTGGCATTTGCTGAAGTGCATTTCCGGTAGAAAACTCCAGTGGTCCCTCACCTGACTGAGT<br>GGCGCAGTCAGAGATAAATGGAACAGAGTCTACAAATAAACATTTTATAAAGATGATCCTTCAATACATATTCTACAGCCCA<br>GTTAAAATGTGAGAGGCCATGATCTACAGACAGCCATTTATAAAGGTGGATCAGGGCCGGACACGGTGGCTCAACGTCTGTA<br>ATCCCGGCACTGTGGGAGGCCAAAGCGGGAGGATCGCTTGAGCCCAGGAGTTCGAGACCAACCTGGCCAACATGCTTTATG<br>TGATTGACACAGACATGGAAGAGATCTGGTCCTTATGAAGAATTCTGTGCTCGCAGGAAAGATCTGTAGGAATAATGAAATG<br>CTTTCTTCATTGATGAGATGAAGTCTCTGTTTTTCAGGCAAGTTGACAGAACAAAACAATGAGAAACGAACTTCTTTAGAAGG<br>TTGAATGAAAGTAAGATCTAGATGAAAAAGTCATTGGCCAAAGATAATTTTTTAAAAAAAACATTCTATGTTAACCCTTGGAA<br>AAAAAAACAAAAAACAACCCAGAGAAAT
miRNA-101-3p FISH Probe(Dig)		TTCAGTTATCACAGTACTGTA

Supplementary Table 2. List of reagents used in this study

Western Blot

Antibody	Company name	Dilution	Catalogue number
METTL3	Abcam	1:1000	Cat#ab19352
FOXG1	abcam	1:1000	Cat#ab196868
MIF	abcam	1:1000	Cat#ab175189
GAPDH	Proteintech	1:1000	Cat#60004-1-1g
LC3 I/II	Cell Signaling Technology	1:1000	Cat#4108S
p62	Cell Signaling Technology	1:1000	Cat#88588S
Anti-mouse IgG-(H+L)	Proteintech	1:2000	Cat# SA00001-1
Anti-rabbit IgG-(H+L)	Proteintech	1:2000	Cat# SA00001-2

Instrument	Company name	Model number
Spark Multimode microplate reader	Thermo Fisher Scientific	MULTSKAN GO
Agilent 2100 Bioanalyzer	Agilent Technologies	G2939B
Leica TCS SP8 confocal microscope	Leica Microsystems	TCS SP8
Chemidoc XRS+ system	BIORAD	Chemidoc XRS+
Gel Doc XR+ System	BIORAD	DIO-RAD GEL DOCXR
Illumina HiSeq X Ten Sequencing System	Illumina	SY-412-1001
Pannoramic digital slide scanner	3DHistech Ltd	P250-018007
AniView100 system	Biolight Biotechnology Co., Ltd.	100

Reagent	Company name	Catalogue number
DMEM	Gibco	Cat#C11995500BT
FBS	Bioexplorer	Cat#BS-1612
Penicillin–streptomycin	Invitrogen	Cat#15140122
Lipofectamine 3000	Invitrogen	Cat#L3000015
Lipofectamine 2000	Invitrogen	Cat#11668019
Opti-MEM medium	Gibco	Cat#31985070
RNAiso Plus	TaKaRa	Cat#9109
PrimeScriptTM RT reagent Kit	TaKaRa	Cat#RR047A
TB Green® Premix Ex TaqTM II	Cell Signaling Technology	Cat#RR820
Hairpin-itTM miRNAs RT-PCR Quantitation Kit	Gene Pharma	Cat#E01006
M-MLV (H-) Reverse Transcriptase	Vazyme	Cat#R021-01
Cytoplasmic extraction kit	Thermo Fisher Scientific	Cat#78833
Cell Counting Assay Kit-8	Dojindo	Cat#CK04
Transwell assay insert	Millipore	Cat#3422
Crystal violet	Baso	Cat#BA4016A
Pierce™ Protein A/G Magnetic Beads	Thermo Fisher Scientific	Cat# 88803
Anti-m6A antibody	Synaptic Systems	Cat# 202003
Anti-rabbit IgG	Sigma-Aldrich	Cat#I8765-5MG
DL500	TaKaRa	Cat#3590A
DAPI	Exonbio	Cat#F-0045
SuperECL Detection Reagent	Yeasen	Cat#36208ES76
RIPA buffer	Thermo Fisher Scientific	Cat#89900
Protease Inhibitor Cocktail	Roche	Cat#4693132001
PVDF	Millipore	Cat#IPVH00010
Pierce BCA Protein Assay Kit	Thermo Fisher Scientific	Cat#23225
dNTP	TaKaRa	Cat#4019
100mM KCl	Sigma-Aldrich	Cat#60135
5mM MgCl2	Sigma-Aldrich	Cat#M1028
10mM HEPES	Solarbio	Cat#H1090
NP-40	Solarbio	Cat#N8030
1mM DTT	Solarbio	Cat#D1070
100 U/ml RNase inhibitor	TaKaRa	Cat#2313A
Normal Rabbit IgG	Cell Signaling Technology	Cat#2729S
Proteinase K buffer	Tiangen	Cat#RT403-02
150 mM NaCl	Sigma-Aldrich	Cat#S6546
RNA pull-down assay kit	Exonbio	Cat#R5126
Dynabead™ MyOne™	Invitrogen	Cat#65001
1M Tris-Cl	Sigma-Aldrich	Cat#T1819
Bafilomycin A1	Ambeed	Cat#A614767
