# Supplementary material for: miR-101/METTL3 axis induces autophagy by interrupting FOXG1/EIF3J-AS1 binding in gliomas
Source: Cell Death Dis. 2025 Dec 13;17(1):99. doi: 10.1038/s41419-025-08285-6 (PMC12830681; doi:10.1038/s41419-025-08285-6)
Supplement: Supplementary file 2 — Supplementary table [file 41419_2025_8285_MOESM2_ESM.pdf]

Figure1-L

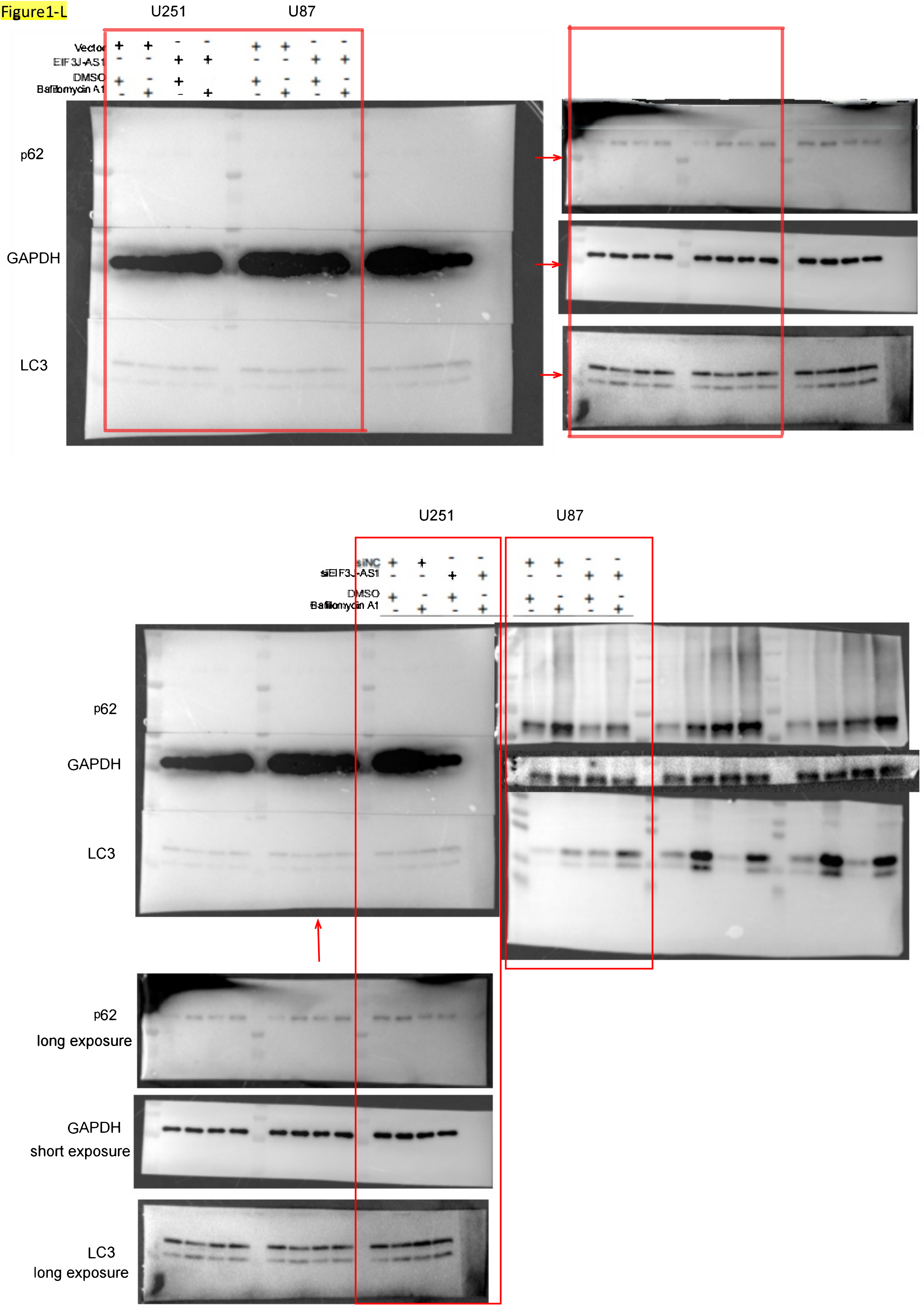
U251
U87
Vector
EIF3J-AS1
DMSO
Bafilomycin A1
p62
GAPDH
LC3
U251
U87
siNC
siEIF3J-AS1
DMSO
Bafilomycin A1
p62
GAPDH
LC3
p62
long exposure
GAPDH
short exposure
LC3
long exposure

Figure2-I

U251

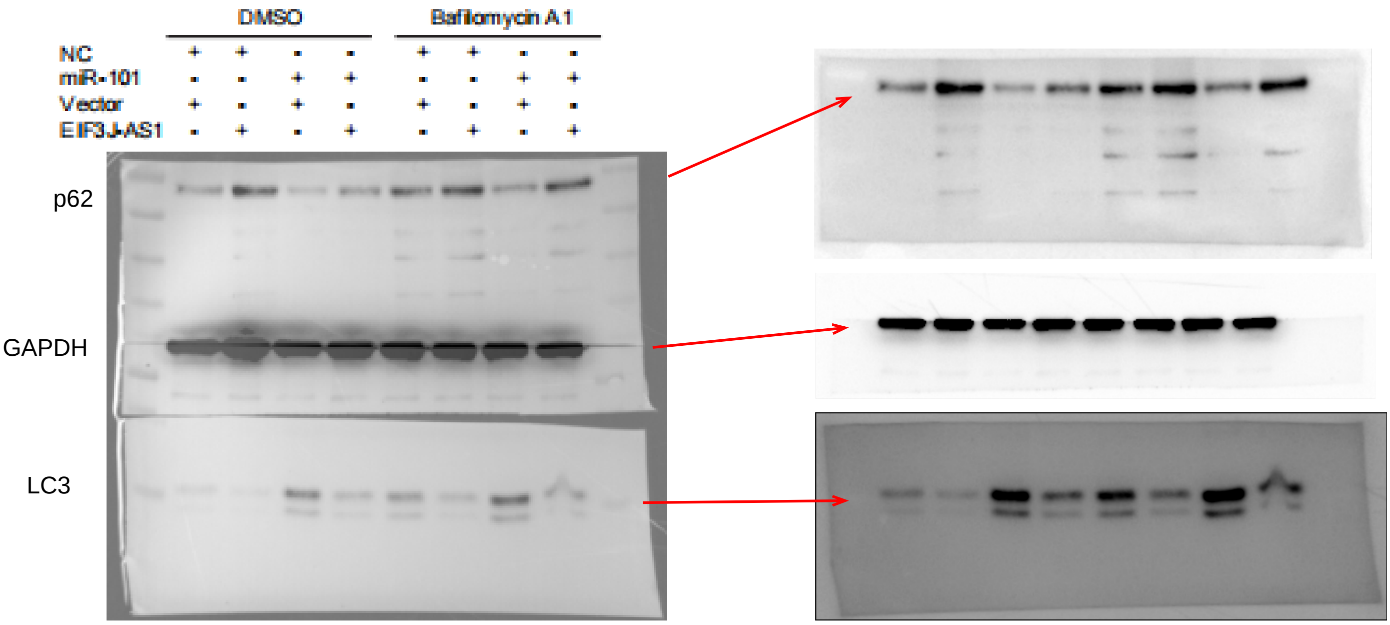

U87

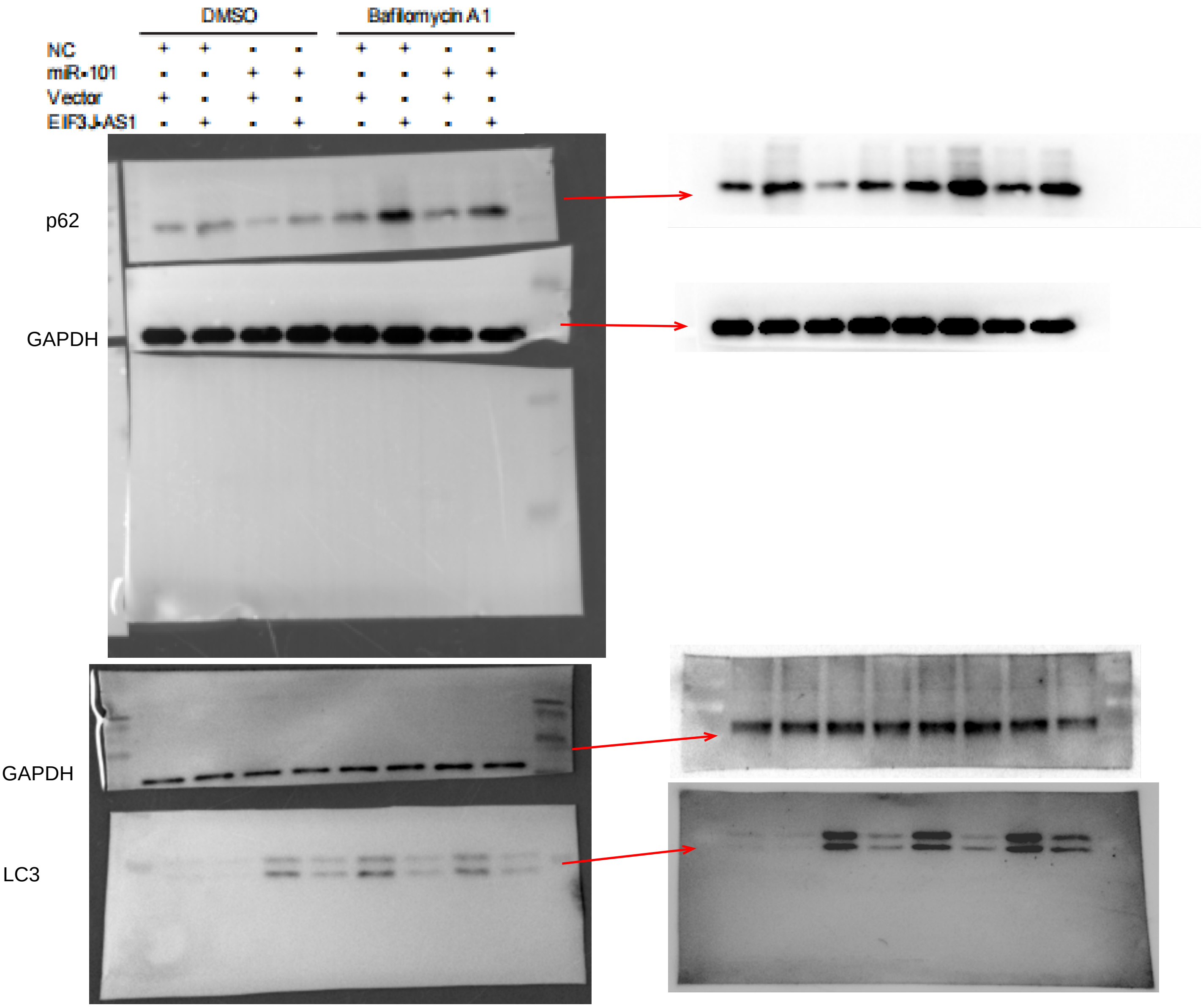

Figure3-C

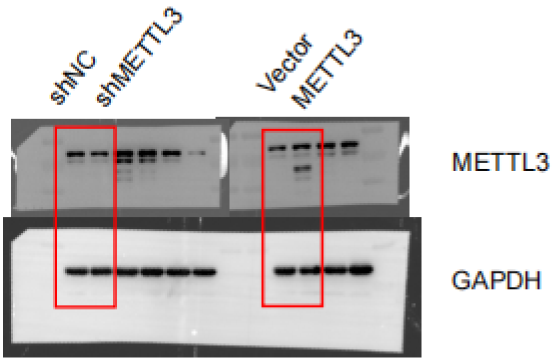

Figure3-H

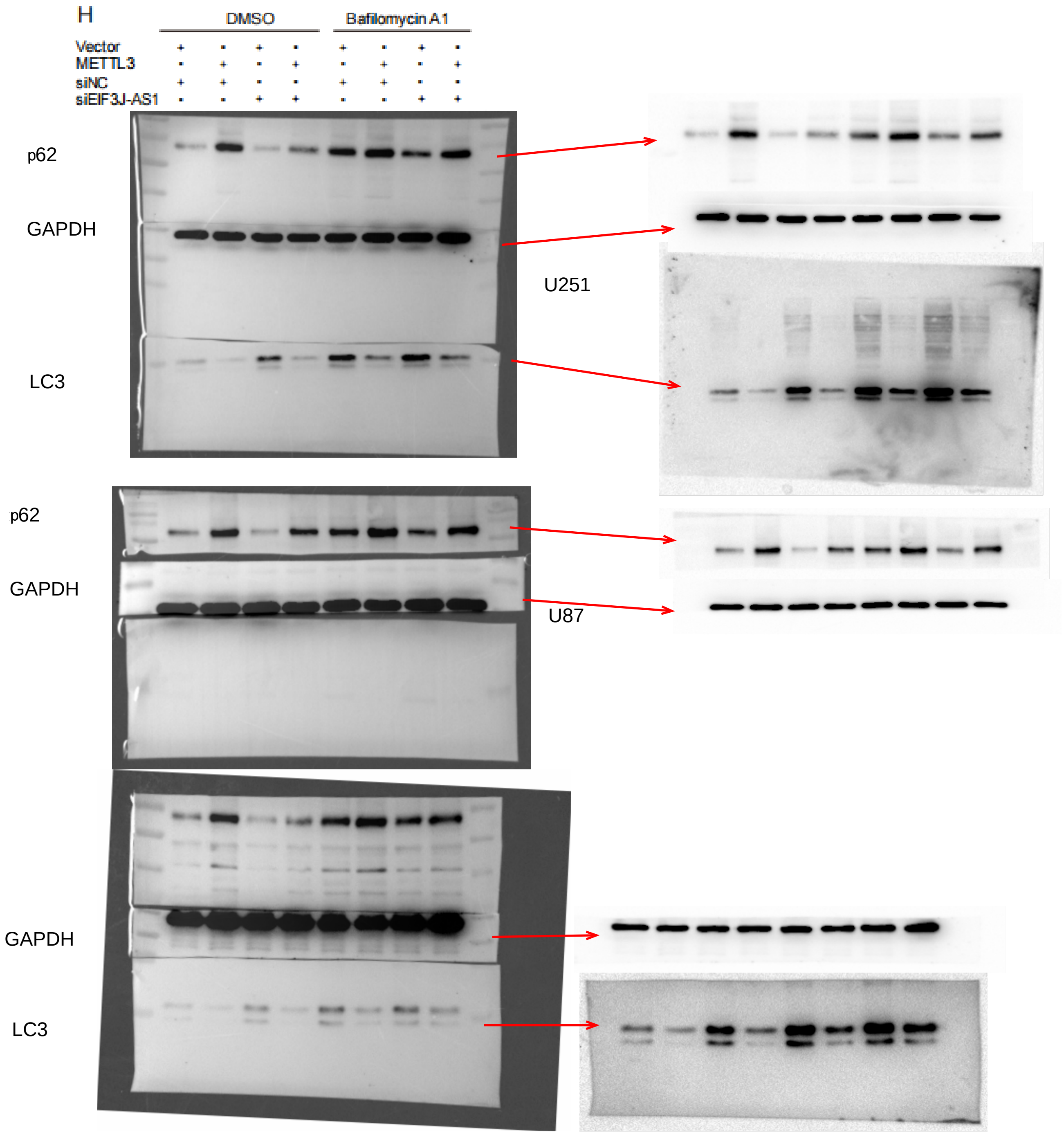

Figure4-I

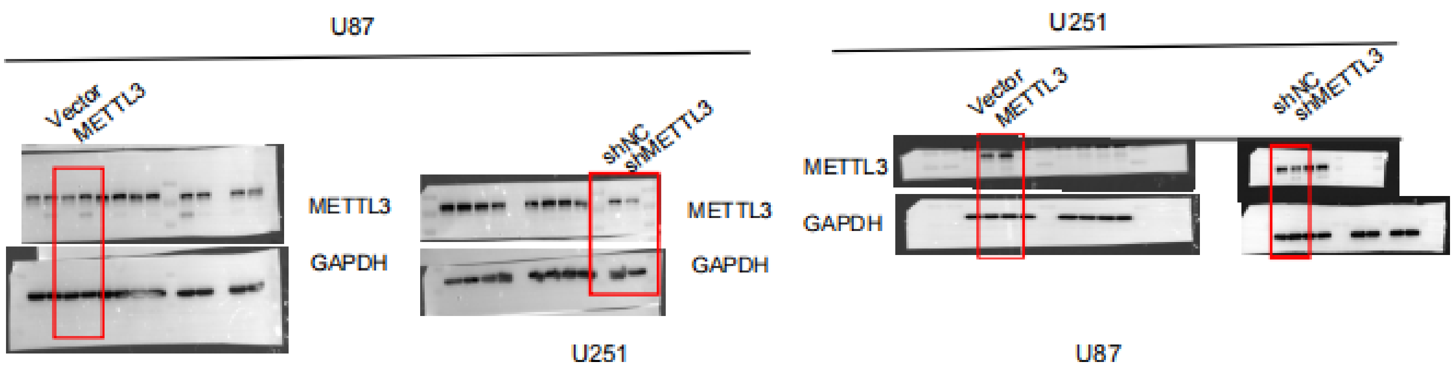
U87
Vector
METTL3
METTL3
GAPDH
shNC
shMETTL3
METTL3
GAPDH
U251
U251
Vector
METTL3
shNC
shMETTL3
METTL3
GAPDH
U87

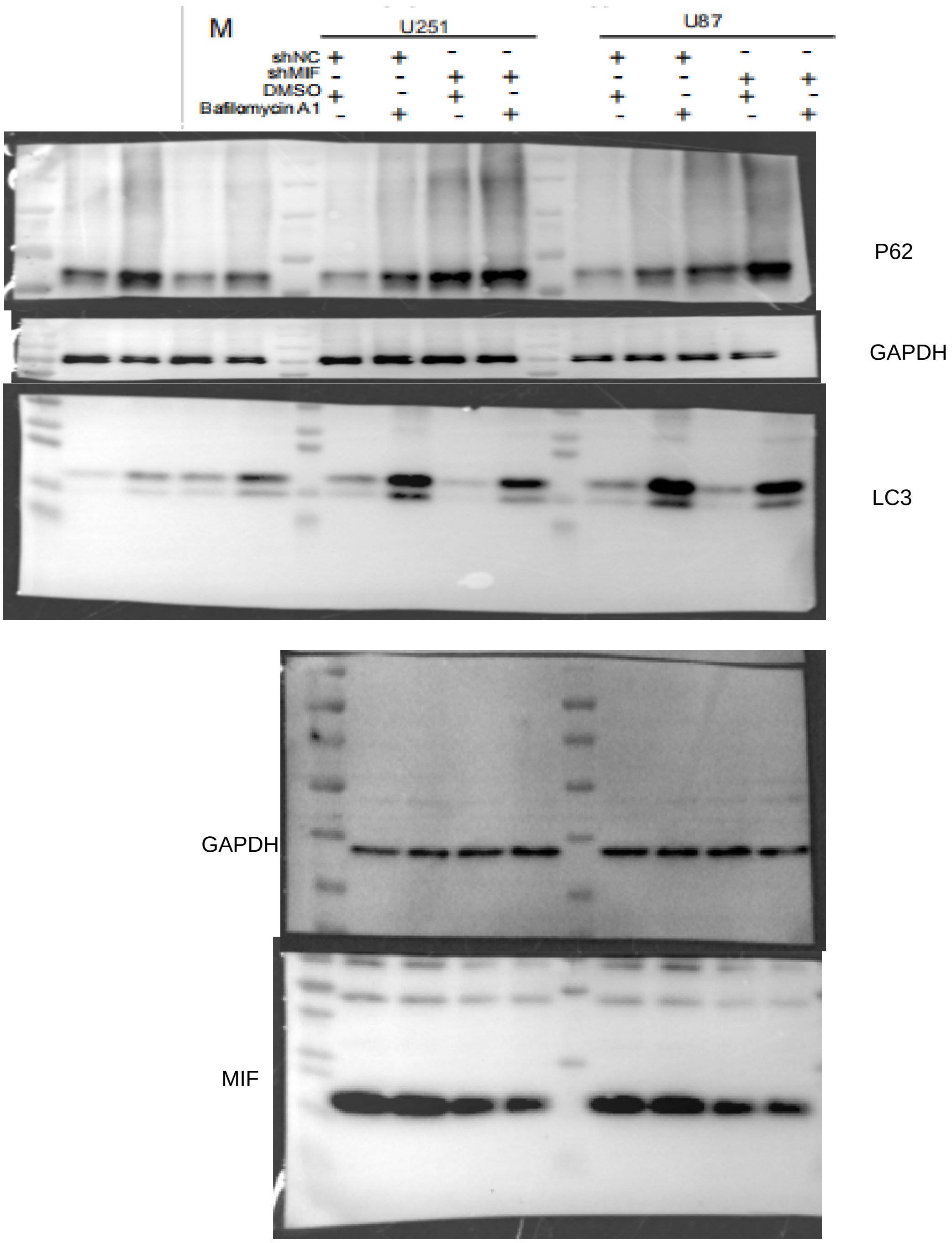
M
U251
U87
shNC + + - - + + - -
shMIF - - + + - - + +
DMSO + - + - + - + -
Bafilomycin A1 - + - + - + - +
P62
GAPDH
LC3
GAPDH
MIF

Figure6

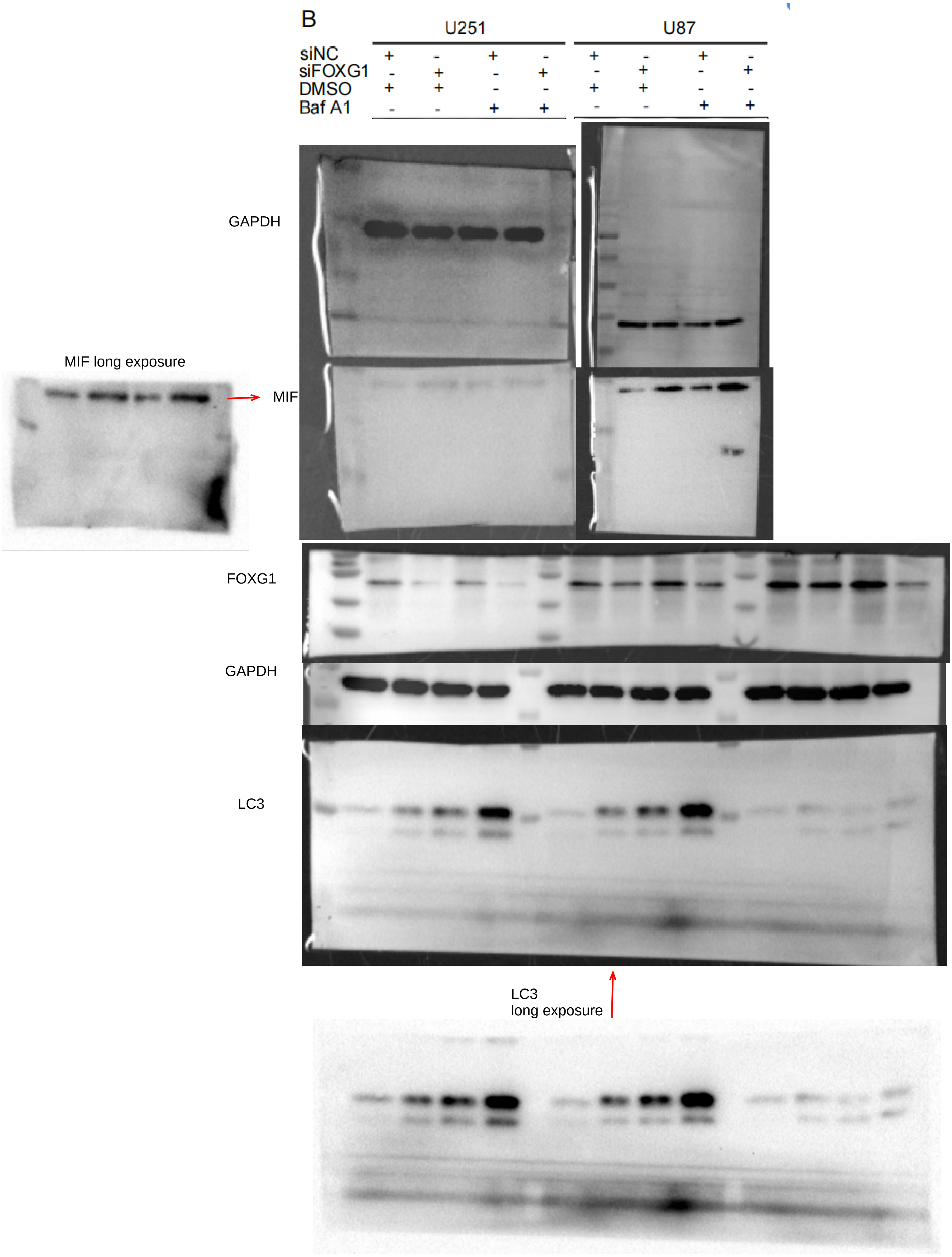
B
U251
U87
siNC + - + - + - + -
siFOXG1 - + - + - + - +
DMSO + + - - + + - -
Baf A1 - - + + - - + +
GAPDH
MIF long exposure
MIF
FOXG1
GAPDH
LC3
LC3
long exposure

Figure6

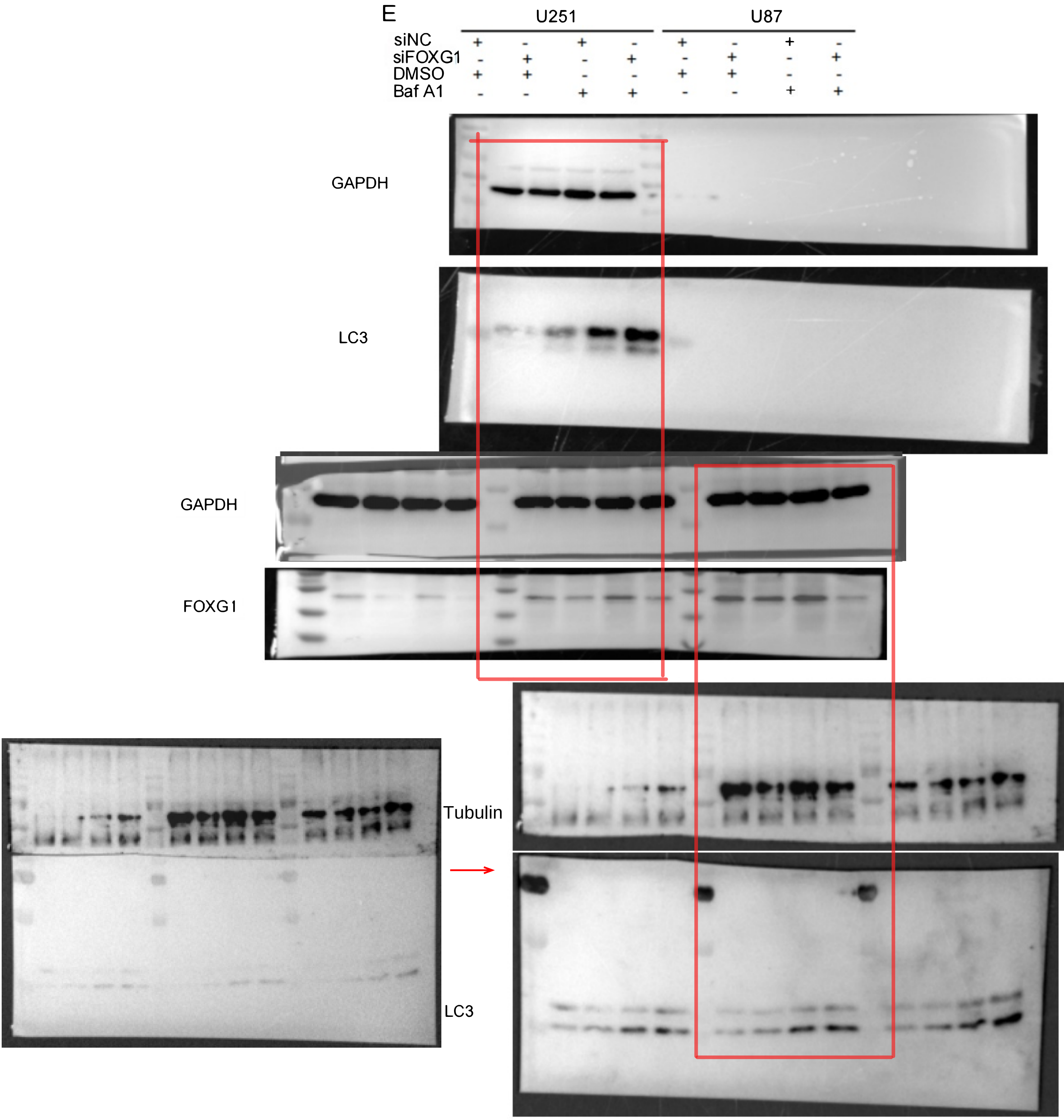
E
U251
U87
siNC + - + - + - + -
siFOXG1 - + - + - + - +
DMSO + + - - + + - -
Baf A1 - - + + - - + +
GAPDH
LC3
GAPDH
FOXG1
Tubulin
LC3

Figure7-C

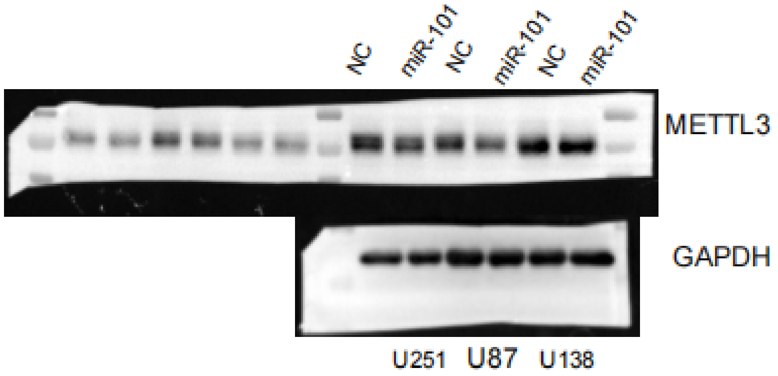
NC
miR-101
NC
miR-101
NC
miR-101
METTL3
GAPDH
U251
U87
U138

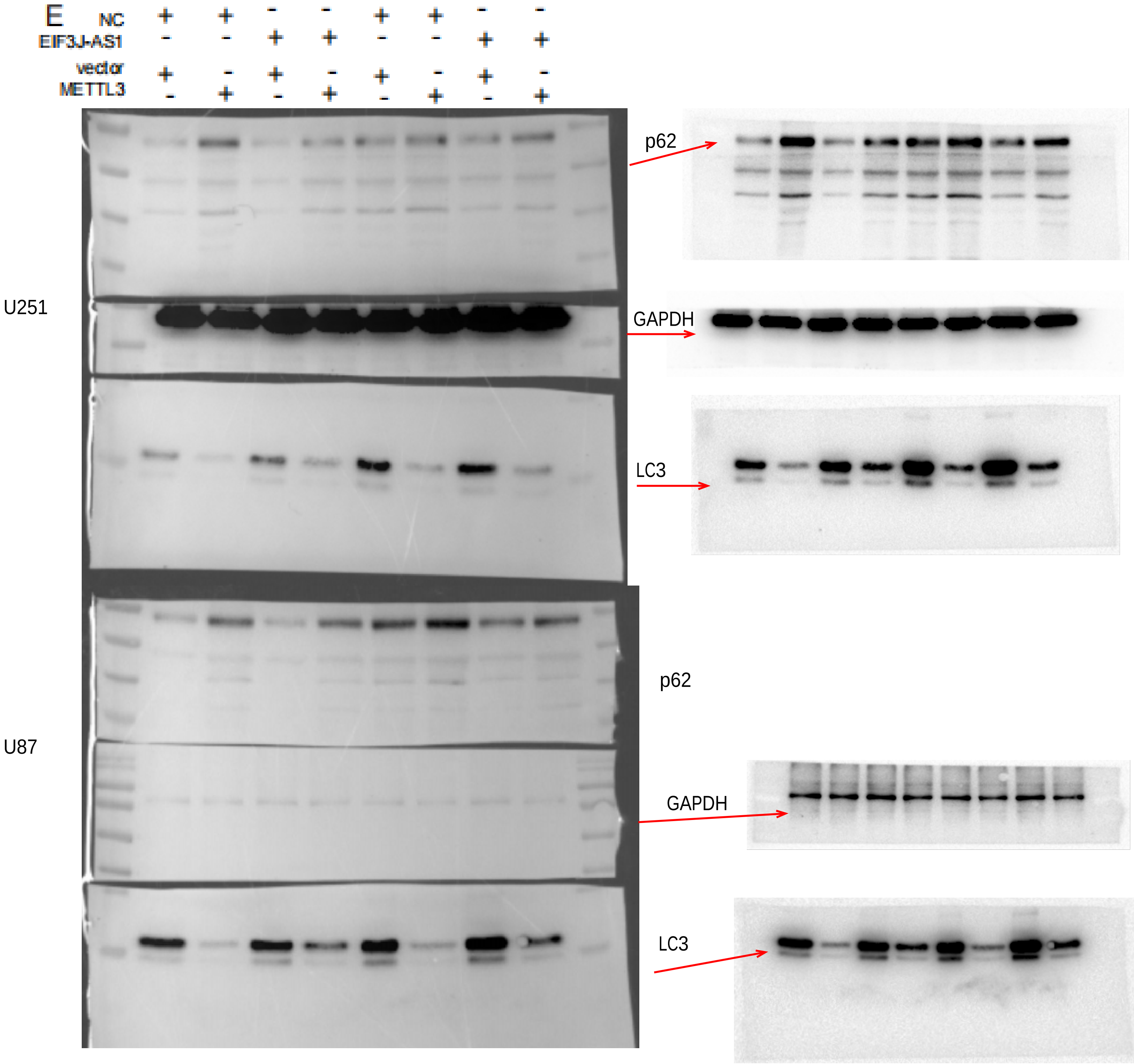
E
NC
EIF3J-AS1
vector
METTL3
U251
p62
GAPDH
LC3
U87
p62
GAPDH
LC3
